# Identification and characterization of early photoreceptor cis-regulatory elements and their relation to Onecut1

**DOI:** 10.1186/s13064-018-0121-x

**Published:** 2018-11-22

**Authors:** Nathalie Jean-Charles, Diego F. Buenaventura, Mark M. Emerson

**Affiliations:** 1 Department of Biology, The City College of New York, City University of New York, New York, NY 10031 USA; 2 Current Address: The Public Health Laboratory, NYC Department of Health and Mental Hygeine, New York, NY 10016 USA; 3 Biology Ph.D. Program, Graduate Center, City University of New York, New York, NY 10031 USA

**Keywords:** Cis-regulatory elements, Rod photoreceptor, Cone photoreceptor, Gene regulatory networks, Retinal development, Flow cytometry, Quantitative analysis

## Abstract

**Background:**

Cone and rod photoreceptors are two of the primary cell types affected in human retinal disease. Potential strategies to combat these diseases are the use of gene therapy to rescue compromised photoreceptors or to generate new functional photoreceptors to replace those lost in the diseased retina. Cis-regulatory elements specific to cones, rods, or both types of photoreceptors are critical components of successful implementation of these two strategies. The purpose of this study was to identify and characterize the cell type specificity and activity of cis-regulatory elements active in developing photoreceptors.

**Methods:**

Cis-regulatory elements were introduced into the developing chicken and mouse retina by electroporation. Characterization of reporter activity in relation with cell type markers was determined using confocal microscopy. In addition, two high-throughput flow cytometry assay were developed to assess whether these elements were downstream of Onecut1 in the photoreceptor specification network.

**Results:**

The majority of cis-regulatory elements were active in both cone and rod photoreceptors and were largely uninfluenced by a Onecut1 dominant-negative construct. Elements associated with the Thrb, Nr2e3, and Rhodopsin genes showed highly enriched activity in cones or rods, and were affected by interference in Onecut1 signaling. Rhodopsin promoter activity was the most highly influenced by Onecut1 activity and its induction could be modulated by the Maf family transcription factor L-Maf. Nr2e3 elements were observed to have activity in cone photoreceptors and Nr2e3 protein was expressed in developing cone photoreceptors, suggesting a role for this predominant rod gene in cone photoreceptor development.

**Conclusions:**

The analysis presented here provides an experimental framework to determine the specificity and strength of photoreceptor elements within specific genetic networks during development. The Onecut1 transcription factor is one such factor that influences the gene regulatory networks specific to cones and rods, but not those that are common to both.

**Electronic supplementary material:**

The online version of this article (10.1186/s13064-018-0121-x) contains supplementary material, which is available to authorized users.

## Background

Photoreceptors are the retinal cells responsible for converting a light signal into a physiological signal used for vision. In almost all vertebrates, these photoreceptors are present as two classes, rods and cones. Rod photoreceptors are responsible for mediating vision under dim light conditions while cone photoreceptors mediate bright light and color vision. The gene regulatory processes at work in these cells is a primary area of investigation due to the highly specialized nature of these cells and the gene expression profiles that underlie this specialization. While many studies have focused on later stages of photoreceptor differentiation when these cells express highly specific transcripts (for instance, phototransduction genes such as opsins, transducing G-proteins, etc), gene regulatory events occurring during the formation of these cells has been investigated to a lesser extent [1–6].

While rod and cone photoreceptors possess highly specific gene expression profiles in the fully differentiated state, these cells also express many of the same genes. These include Otx2 (necessary for photoreceptor cell formation), Crx (required for differentiation), and retinol binding proteins (photoreceptor function), among many others [7–10]. This suggests that these cells may have shared gene regulatory mechanisms as well as divergent ones. This makes sense due to the similar functional responsibilities of these cells as photoreceptors, but may also be due to these cells sharing an evolutionary history as sister cell types as well as a possible shared developmental history [11].

Nrl is one of the key transcription factors participating in the gene regulatory network of rod photoreceptors [12]. In the mature retina, Nrl expression is highly specific to rod and not cone photoreceptors [13]. Mouse knockout data supports a functional role for Nrl in promoting the expression of rod-specific genes such as rhodopsin while repressing the expression of cone-specific genes such as S-opsin and cone phototransduction components [1, 12, 14]. Part of this effect is believed to be mediated by the transcription factor Nr2e3, which is specifically expressed in rods in the mature retina and produces a loss-of-function phenotype that partially phenocopies that of Nrl [15–17]. In addition, ectopic expression of Nrl in postmitotic cones leads to suppression of cone genes and activation of rod genes [18]. These data among others has led to the common photoreceptor precursor model, in which postmitotic photoreceptors are formed during development and these cells become rods if they express Nrl and cones if they do not express Nrl [19].

Recent findings have also identified a role for the Onecut1 and Onecut2 family members in early regulatory events in cone and rod genesis [20]. Mouse knockouts of these genes lead to upregulation of Nrl transcripts during embryonic retinogenesis, presumably underlying the formation of precocious rods [20, 21]. These changes occur concomitantly with a decrease in the cone-associated genes Rxrg and Thrb, suggesting that these precocious rods might arise at the expense of cone photoreceptors [20, 21]. In the chicken retina, introduction of a dominant-negative Onecut1 construct leads to upregulation of L-Maf, the functional equivalent of Nrl in the chicken retina, as well as a rhodopsin reporter element [20]. Misexpression of OC1 in the mouse postnatal Day 0 (P0) retina leads to induction of early cone genesis markers (Thrb and Rxrg) and suppression of Nr2e3 in mature rods [20]. Taken together, these data suggest that OC1 and OC2 act upstream of Nrl to prevent the formation of rods and promote cone transcriptional programs. In addition, OC1 is active in retinal progenitor cells (RPCs), suggesting that the decision to form a cone or rod could be ultimately controlled in a RPC and not a postmitotic precursor [20].

Here, we characterize the activity of known and novel cis-regulatory elements active in developing photoreceptors. The activity of these elements in rods and cones and other retinal cell types was quantitatively assessed in both chick and mouse species. To place these elements in the Onecut1 pathway, the activity of these elements in response to rod-inducing Onecut1 dominant-negative constructs were quantitatively measured using a high-throughput flow cytometry assay. These results provide an experimental framework to define the regulatory networks active in cone and rod genesis. In addition, they suggest that Nr2e3 is involved in the early gene regulatory networks of both cones and rods.

## Methods

### Animals

All procedures involving animals were approved and conducted in accordance to the City College of New York Institutional Animal Care and Use Committee. CD-1 mice and fertilized chicken eggs were obtained from Charles River.

### Cloning and DNA electroporation

Evolutionarily conserved elements were identified using the Evolutionary Conserved Regions (ECR) browser and University of California Santa Cruz (UCSC) genome browser. ThrbCRM1::GFP, ThrbCRM1::AU1, ThrbCRM2::GFP, Rbp3Enh1::GFP, CAG::OC1EnR, and CAG::EnR [22] and Chx10BPEnh::GFP and cow Rhodopsin::GFP constructs [22] have been previously described. The Nrl::GFP reporter construct was made using Sal1/EcoR1 to transfer a 3.2 kb mouse Nrl promoter fragment [23] into Stagia3 [24]. All other elements were PCR amplified from mouse (C57Bl6) or chicken (White leghorn) genomic DNA using Herculase polymerase (Stratagene). Amplicons were cloned into PGemTeasy (Promega) and subcloned into Stagia3 using either EcoR1 or reamplified with primers with additional Sal1 and Xho1 sites (CrxEnh2, Rbp3Enh2). Elements were tested in both Stagia3 orientations and in general the strongest orientation was used for all further experiments. All constructs generated through polymerase chain reaction (PCR) amplification were sequence verified. The genomic coordinates of the cis-regulatory elements used in this study can be found in Additional file 1: Table S1. Rhodopsin::TdTomato was made by PCR amplifying TdTomato using primers with Age1 and BsrG1 sites and cloned into Stagia3 cut with these enzymes, replacing EGFP with TdTomato. The cow Rhodopsin element [23] was inserted using Age1/Xho1. An L-Maf cDNA was PCR amplified (see Additional file 2: Table S2 for primers) from late chick embryonic cDNA using Herculase polymerase and cloned into PGemTeasy. This was cloned into a pCAG vector using EcoR1. A L-Maf-EnR fusion misexpression plasmid was constructed using the primers listed in Additional file 2: Table S2 and a PCR-based stitching protocol described previously [20].

### Electroporation

All experiments were performed as previously reported with the exception that a Nepagene electroporator was used [22]. The electroporation chamber was rinsed with 70 μl of 1XPhophate Buffered Saline for a minimum of 8X.

### In situ hybridization

RNA in situ hybridization was performed as previously described [25]. A portion of the chick Nr2e3 3′ untranslated region was PCR amplified (see Additional file 2: Table S2), cloned into PGem-T Easy (Promega, A1360) and sequence verified. A second round of PCR was performed to generate a template with a T7 polymerase site at the 3′ end of Nr2e3 and an antisense digoxigenin probe was generated using T7 polymerase in an in vitro transcription reaction.

### Immunofluorescence

Primary antibodies used were chicken anti-GFP (ab13970, Abcam, 1:2000), rabbit anti-GFP (A-6455, Invitrogen, 1:500) mouse anti-ß-galactosidase (40-1a-s, DSHB, 1:20), chicken anti-ß-galactosidase (ab9361, Abcam, 1:1000) mouse anti-Visinin (7G4-s, DSHB, 1:250), mouse anti-Pax6 (Pax6-s, DSHB, 1:20), rabbit anti-PKC-alpha (P4334, Sigma, 1:500), mouse anti-AU1 (AU1, Biolegend, 1:1000) rabbit anti-MafA/L-Maf (gift from Celio Pouponnot, 1:500) [26], rabbit anti-Rxrg (ab15518, Abcam, 1:500), mouse anti-Rxrg (Santa Cruz Biotechnology SC-365252, 1:50), rabbit anti-cone arrestin (Millipore, AB15282, 1:2000), mouse anti-Nr2e3/PNR (R&D Systems, PP-H7223–00, 1:250). All secondary antibodies were obtained from Jackson Immunoresearch and were designated as appropriate for multiple labeling. Retinas were processed for staining as previously described [22].

### Microscopy

All Confocal images were obtained using a Zeiss 710 confocal except for those in Fig. 4, which used a Zeiss 880. Images were analyzed using Zen and Image J software.

### Retina dissociation and flow cytometry

For dissociation, retinas were removed from culture filters and several portions were removed by dissection: remaining retinal pigmented epithelium, a portion of unelectroporated retina near the ciliary margin and condensed vitreal matter were removed in HBSS (GIBCO, 14170112), dissociated using papain (Worthington, L5003126) and DNASE 1 (experiments were performed with both Sigma-Aldrich 4,716,728,001 or 4,536,282,001, with no observed difference). Cells were fixed in 4% paraformaldehyde, washed 3X in 1XPBS and filtered using 40 μm cell strainers (Biologix, 15–1040) into 4 ml tubes (BD Falcon, 352,054). For every flow cytometry experiment, three control retinas (non-electroporated, electroporated with CAG::GFP, electroporated with UbiquitinC::TdTomato) were used to generate compensation controls and to define single-positive and double-positive populations. Cells were analyzed using a BD FACS Aria flow cytometer with a 488 laser and FITC and PE filters. All flow cytometry experiments were replicated in two independent experiments using > 3 biological replicates in each experiment. Selected flow cytometry plots shown in figures were manually adjusted using the Biexponential Tool in FACS Diva software to standardize plots for visualization purposes in the figures (denoted by a blue “M” next to the plot).

### Statistical analysis

One-way ANOVAs with a post hoc Dunnett’s test were calculated using R 3.3.0. [27] and the multcomp package [28]. Independent t-tests were run using JASP software [29].

## Results

### Cis-regulatory element identification

**Fig. 1:**
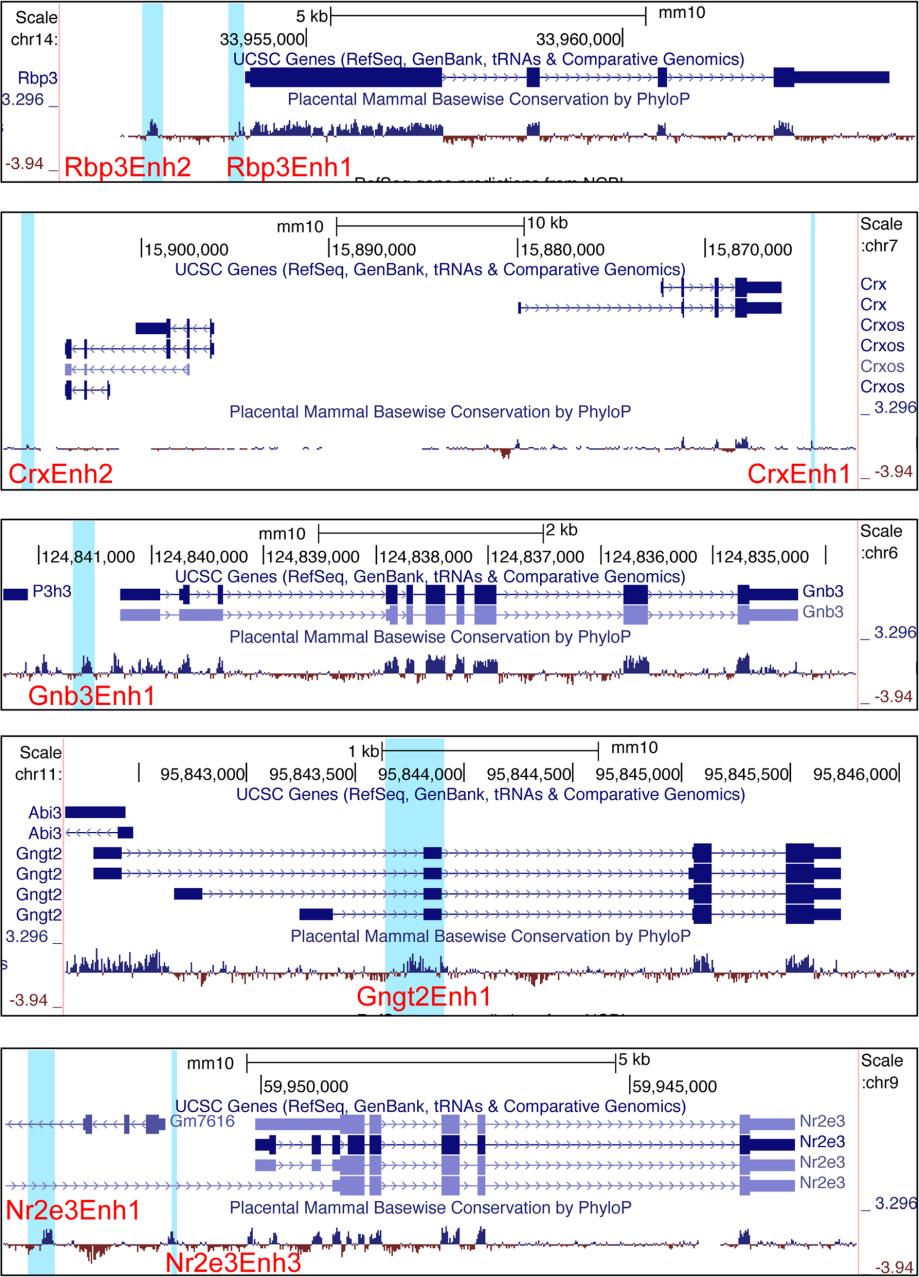
Mouse genomic location of potential regulatory elements. Snapshots of UCSC genome browser showing location of regulatory elements relative to their associated genes, shown by the included Ref-Seq tracks. Placental mammalian conservation tracks are shown below. Cis-regulatory elements are identified by red font and the location is shown with light blue shading

A number of previously reported elements were identified and cloned into the Stop TAta eGfp Ires Ap version 3 (Stagia3) reporter vector, which is an effective reporter vector for both the developing chicken and mouse retina [22, 24]. These included elements associated with genes that in the adult retina are expressed in all photoreceptors (Rbp3, Crx), only in rods (Nrl, Nr2e3, Rhodopsin) or only in cones (Thrb) (Table 1) [1, 20, 23, 30–32]. In addition, new candidate elements located near known photoreceptor genes were identified (bold lettering in Table 1 labels new elements) based on the criteria of being evolutionarily conserved and tested for enhancer activity. These included elements located in proximity to Nr2e3, Gnb3, Gngt2, Crx, and Rbp3. The CrxEnh2 element was identified based on sequence similarity to conserved sequence elements of CrxEnh1 and not because of evolutionary conservation (Additional file 3A). In addition, an analysis of data from previously reported chromatin immunoprecipitation data determined that Nrl protein occupied both CrxEnh1 and CrxEnh2 (Additional file 3B) [33]. This suggests that the CrxEnh2 element is likely a bona fide cis-regulatory element active in the retina. The genomic locations of these Crx-associated elements are shown in Fig. 1. Previously identified elements were given specific names here for the purposes of distinguishing them from new elements. All elements were initially identified as positive for alkaline phosphatase reporter activity in chick embryonic day 5 (E5) retinas, at levels above that observed with a Stagia3 plasmid without additional cis-regulatory elements [22](data not shown).

**Table 1:** Photoreceptor cis-regulatory elements used in this study

Cones + Rods	Rods	Cones
mRbp3Enh1	cowRhodopsin	cThrbCRM1
**mRbp3Enh2**	mNrl Promoter	cThrbCRM2
mCrxEnh1	mNr2e3Enh1	**mGnb3Enh1**
**mCrxEnh2**	**cNr2e3Enh2**	**mGngt2Enh1**
	**cNr2e3Enh3**	

Cis-regulatory elements that have not been previously reported are shown in bold

**Fig. 2:**
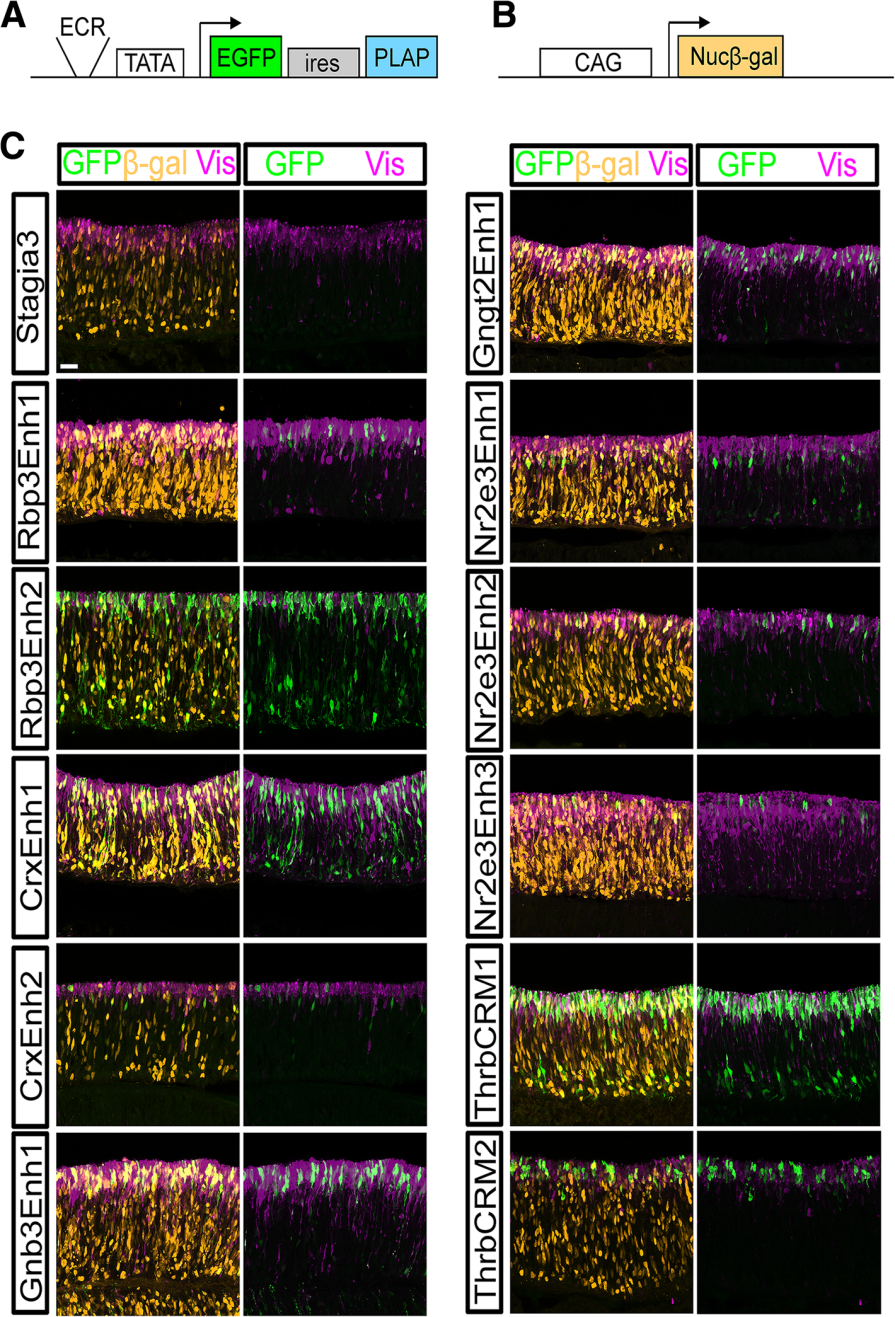
Activity of GFP reporters relative to photoreceptors in the chicken retina. **a** Schematic of the Stagia3 reporter vector Arrow represents transcription initiation position and direction. **b** Schematic of the Nucβ-gal driven by the CAG promoter element co-electroporation control. **c** E5 Retinas electroporated with a CAG::Nucβ-gal and the GFP reporter shown to the left of panels, explanted and cultured for 2 days, and imaged by confocal microscopy for the expression of GFP (green, rabbit antibody), Nuc β-gal (orange, chicken antibody), and Visinin (purple). The scleral portion of the retina is located near the top of the image. Scale bar in top left panel represents 20 μm and applies to all panels. Abbreviations: ECR (Evolutionarily Conserved Region) TATA (TATA box), EGFP (Enhanced Green Fluorescent Protein), ires (Internal Ribosomal Entry Site), PLAP (Placental Alkaline Phosphatase), Nucβ-gal (Nuclear β-galactosidase)

The activity of elements was assessed first in the developing chicken retina through electroporation of embryonic day 5 (E5) retinas, explanting for 2 days and then harvesting. During this time it is expected that cone photoreceptors are generated and rod photoreceptor genesis has not begun, as determined previously by expression onset at E9 of L-Maf/MafA (referred to hereafter as L-Maf), the earliest known rod marker in chickens [34, 35]. To determine if any of these elements were capable of driving a reporter in a photoreceptor-like pattern, retinas electroporated with Stagia3 reporter plasmids were examined for Green Fluorescent Protein (GFP) expression in relation to visinin (a photoreceptor marker at this developmental time) and Pax6 (a marker of RPCs, amacrine cells, horizontal cells, retinal ganglion cells, and Mueller Glia). Representative confocal images are shown in Fig. 2 and Additional file 4.

As previously reported, a Stagia3 vector lacking cis-regulatory elements drives GFP in very few cells (Fig. 2). In contrast, the four elements associated with the Crx and Rbp3 genes, which are expressed in both cone and rod photoreceptors, showed robust GFP expression in electroporated visinin-positive photoreceptors. In addition, elements associated with cone genes (Thrb, Gngt2, Gnb3) were also active in visinin-positive cells, as expected. For elements associated with rod-enriched genes, there was some divergence from expected results. Very few rod photoreceptors are likely to be present at this embryonic timepoint given the lack of L-Maf positive cells before embryonic day 9 [35]. The cow Rhodopsin element has previously been shown to have a very low activity level in the chicken retina at this timepoint, ([20] and see also Fig. 5). Three elements located proximal to the Nr2e3 gene were tested. These included one element previously characterized in the mouse retina (mNr2e3Enh1) [1]. The second element (cNr2e3Enh2) is a chicken DNA sequence that has some similarity with mNr2e3Enh1, and may be the homolog of mNr2e3Enh1. cNr2e3Enh3 is another chicken element that is conserved in mice but that has not been previously characterized. Somewhat surprisingly, all three of these Nr2e3 elements were active in Visinin-positive cells that were likely cone photoreceptors. Previously, it has been reported that Nr2e3 is also transiently expressed in early stage cone photoreceptors in the zebrafish retina and at one timepoint in the mouse [16, 36]. Thus, it is possible that this observed expression in these presumptive cone photoreceptors is recapitulating the normal Nr2e3 expression in cones. In support of this hypothesis, a recent transcriptomic analysis of the cone associated reporter ThrbCRM1 revealed the Nr2e3 gene to be one of the most differentially expressed genes in the ThrbCRM1 active population [37]. To further confirm if Nr2e3 is expressed during this phase of photoreceptor genesis when cone cells are born but rods are not, an RNA in situ hybridization was performed on E6 retinas, a timepoint several days before the first reported rod photoreceptors. This revealed Nr2e3-positive cells located along the scleral surface where newborn photoreceptors would be expected to be found (Additional file 5) [35]. As additional confirmation that these GFP-positive cells were cone photoreceptors, an antibody to Rxrg was used. Rxrg has been characterized as an early cone photoreceptor gene in mammals and is expressed in a subset of chicken cones [38, 39]. Rxrg and GFP double-positive cells were found in retinas electroporated with each of the three Nr2e3::GFP reporters, confirming that some of the cells with active Nr2e3 elements are cone photoreceptors (Additional file 6). Sections of retinas electroporated with the set of enhancers and stained with Pax6 revealed qualitatively less expression of GFP driven by all of the elements in the Pax6-positive population (Additional file 4). However, there were cells present for several of the elements outside of the photoreceptor layer which could represent activity in RPCs that generate photoreceptors and other cells, as in the case of ThrbCRM1, and/or there is some expression in some non-photoreceptor cells. Thus, this analysis identifies all of these cis-regulatory elements as qualitatively enriched in activity in developing cone photoreceptors.

### Assessment of Cis-regulatory activity in mouse rod photoreceptors

**Fig. 3:**
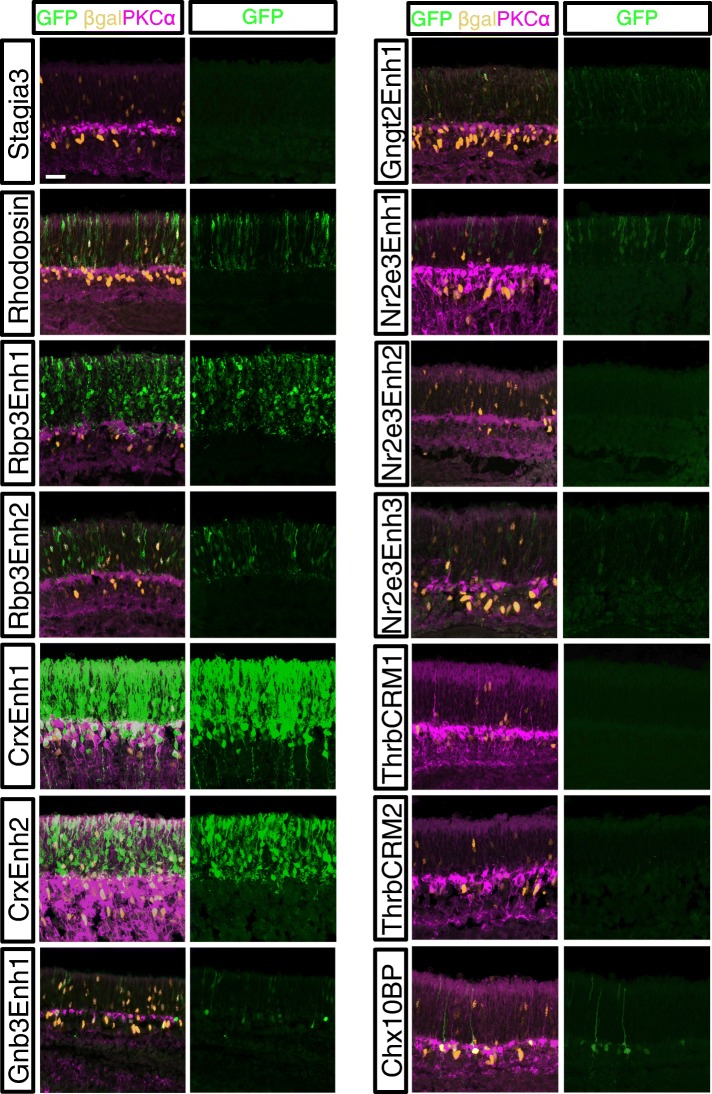
Activity of GFP reporters in the mouse postnatal retina. Retinas electroporated with a Cag::Nucβ-gal and the GFP reporter shown to the left of panels and imaged by confocal microscopy for the expression of GFP (green, chicken antibody), Nucβ-gal (orange, mouse antibody), and PKCalpha (purple). The scleral portion of the retina is located near the top of the image. Scale bar in top left panel represents 20 μm and applies to all panels

The activity of these same elements was assessed in mouse rod photoreceptors. Electroporation at postnatal day 0 (P0) allows for plasmid targeting to rod photoreceptors, bipolar cells, Mueller glia, and amacrine cells, but does not efficiently target cone photoreceptors, retinal ganglion cells, or horizontal cells, which are formed earlier in retinal development. P0 mouse retinas were electroporated ex vivo with the Stagia3 reporter vectors and a co-electroporation control and cultured for 8 days to allow for the formation of an outer nuclear layer (ONL) and inner nuclear layer (INL). Retinas were imaged for GFP, the co-electroporation control, and PKCalpha, to aid in identification of the ONL/INL boundary and also to allow for the positive identification of rod bipolar cells. As has been previously observed, activity of the CAG element in ex vivo mouse preparations was relatively strong in the INL as compared to the ONL [1, 22]. Similar to the chicken retina, the Stagia3 vector has low background activity in the mouse retina, as was previously reported (Fig. 3). Elements associated with genes expressed in both cone and rod photoreceptors were observed to have activity in rod photoreceptors. These included the two elements associated with Crx as well as the two elements associated with Rbp3. In addition to rod photoreceptor expression, reporter activity driven by CrxEnh1 was also detected in the INL in cells with the morphology of bipolar cells. As Crx is expressed in bipolar cells, this may reflect the activity of this element in normally promoting Crx expression in both photoreceptors and bipolar cells. For the cone photoreceptor gene elements, it was expected that elements that were active in cones, but not rods, would have no activity in the P0 retina. The ThrbCRM1 reporter, that was previously identified as specific to RPCs that preferentially generates cone photoreceptors and horizontal cells, does not label cells in mouse retinas electroporated at P0. The ThrbCRM2 element was also inactive, suggesting that this element could be either cone-specific, not active at all in the mouse retina, or too weak to detect in this assay. The elements associated with the Gngt2 and Gnb3 gene were found to be active in the ONL indicating that they are also active in rod photoreceptors. Therefore, these elements do not recapitulate the restricted expression of the Gngt2 and Gnb3 genes observed in adult mouse cone photoreceptors using immunofluorescence [40, 41]. Previous studies have determined that P0 in vivo electroporation of the mouse retina does not efficiently target cone photoreceptors [20, 42]. However, this has not been quantified in ex vivo preparations as were used in this study. To determine if cone photoreceptors were targeted, P0 retinas were co-electroporated with CAG::GFP and the strong photoreceptor reporter Rbp3Enh1::GFP and retinas were cultured for 8 days. Retinal sections were processed to detect GFP and the two cone markers Rxrg and cone arrestin. Examination of GFP-positive cells in the ONL revealed that only a small percentage of GFP-positive cells appeared to be positive for Rxrg (Additional file 7). From this experiment, we conclude that the robust photoreceptor activity we observe with the elements in this study was unlikely to be a result of targeting cone photoreceptors, and therefore the activity observed was mainly rod photoreceptors. The Nrl and rhodopsin elements have previously been reported to have rod activity in an electroporation assay and the specificity of this activity was shown in this study [1, 23]. The previously identified mNr2e3Enh1 element was active in rods, however the cNr2e3Enh2 element did not have detectable GFP activity. The cNr2e3Enh3 element was active in rod photoreceptors to a similar extent as the mNr2e3Enh1 element. Overall, this analysis characterized nine different elements that drive GFP reporter activity in developing mouse photoreceptors.

**Fig. 4:**
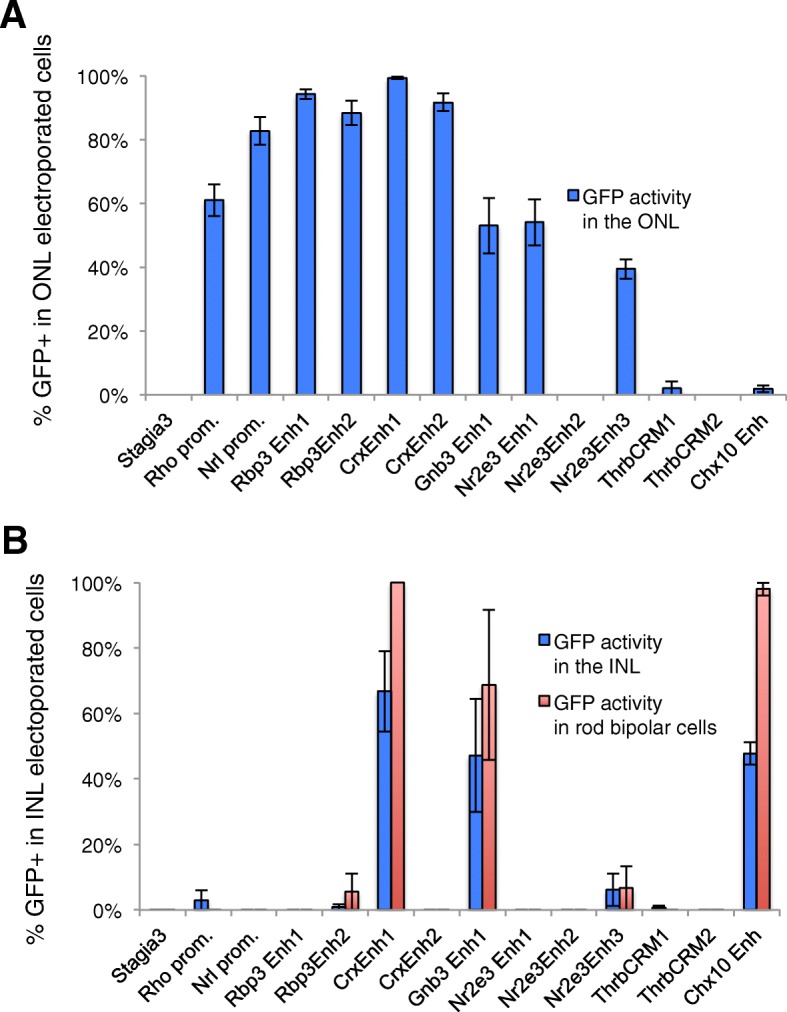
Quantitation of reporter activity in the mouse postnatal retina. Fluorescent cells from confocal images as shown in Fig. 3 were quantified using ImageJ. All electroporated cells (either β-gal-positive or GFP-positive) were identified and categorized as having their cell body located in either the ONL or the INL. For those cells located in the INL, cells were also examined for expression of PKCalpha, a rod bipolar marker. **a** The percentage of GFP-positive cells out of all electroporated cells is plotted with blue bars. **b** The percentage of GFP-positive cells out of all electroporated cells in the INL is plotted with blue bars. The percentage of GFP-positive cells that were identified as rod bipolar cells is shown with red bars

The specificity of these elements for rod photoreceptors was also determined by quantifying the number of reporter-positive rod photoreceptors relative to the electroporated population (Fig. 4a). While the Stagia3 vector without any enhancer elements has very little basal reporter expression, most of the elements drove GFP reporter expression in 50% or more of the electroporated rod photoreceptor population. In contrast, the cNr2e3Enh2 element and the two Thrb elements were not active in rod photoreceptors, similar to the basal level of the Stagia3 vector by itself and the Chx10 bipolar element. We next assessed whether any of the elements drove reporter expression in electroporated INL cells. The Chx10 bipolar element served as a positive control based on a previous report and indeed had positive activity in INL cells (Fig. 4b) [22]. Co-labeling with PKCalpha confirmed that at least some of the GFP-labeled cells were bipolar cells and that nearly all of these rod bipolar cells were labeled by the Chx10 reporter. Out of all the active photoreceptor elements, only the CrxEnh1 and Gnb3Enh1 elements were also active in a substantial number of INL cells (Fig. 4b). Like the Chx10 element, nearly all of the electroporated rod bipolar cells were positive for the CrxEnh1 driven reporter. This observed bipolar activity was not previously reported for this element in its initial characterization [1]. Overall, this quantitative analysis in the mouse postnatal retina allows the classification of these cis-regulatory elements into three groups: rod-photoreceptor specific, rod photoreceptor and bipolar active, and inactive.

### Onecut1 dominant-negative induction of rhodopsin

**Fig. 5:**
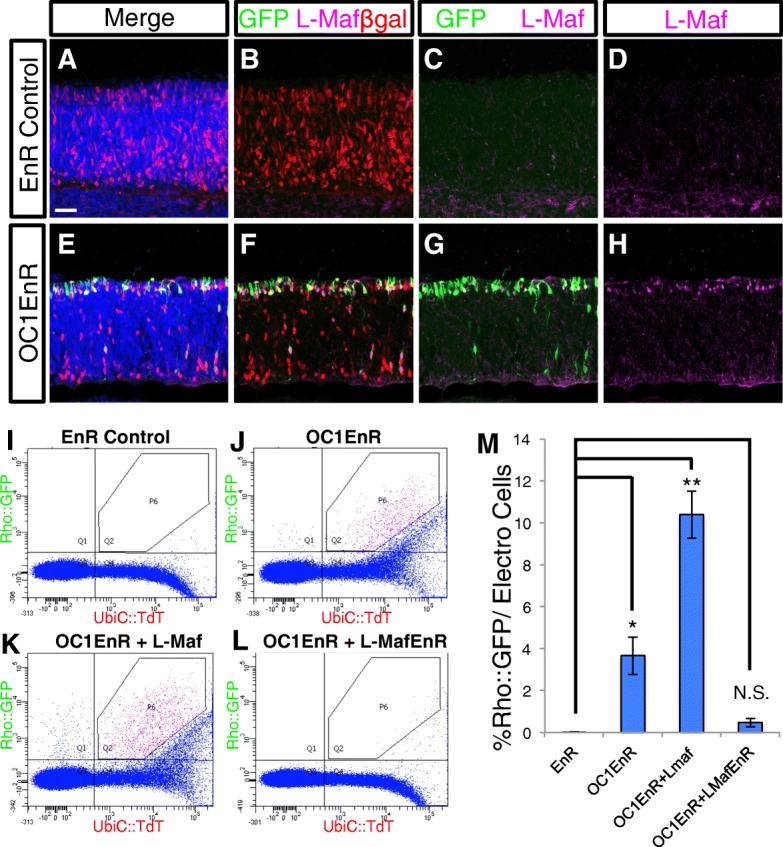
Onecut1 dominant-negative induces L-Maf expression and a Rhodopsin reporter in the chicken retina, and is modulated by L-Maf activity. **a**-**h** Confocal imaged sections of retinas electroporated with CAG::Nuc β-gal, Rhodopsin::GFP, and either CAG::EnR (**a**-**d**) or CAG::OC1EnR (E-H). Retinas were immunostained with antibodies to GFP (green, chicken antibody), β-gal (red, mouse antibody), and L-Maf (magenta) and nuclei were stained with DAPI (blue). **i**-**l** Representative flow cytometry plots of dissociated cells from retinas electroporated with UbiC::TdT and Rhodopsin::GFP and the CAG construct(s) shown above each plot. The P6 gate demarks unambiguous double-positive cells. M) A bar graph of the percentage of Rho::GFP-positive cells in the electroporated population of retinas that were coelectroporated with the CAG construct(s) shown along the x-axis. Error bars represent standard error of the mean. Statistical analysis using Dunnett’s test for comparison of EnR group to 3 experimental groups. Significance value denoted by * = 0.1, ** = < 0.01 and N.S. signifies “No Significance”. *N* = 4 biological replicates for each condition. Scale bar in A represents 20 μm and applies to all image panels

Previous studies have suggested a role for Onecut1 in repressing rod genes such as Nrl/L-Maf in both mouse and chicken while promoting the expression of cone associated genes such as Thrb and Rxrg [20, 21]. This suggests that Onecut1 may play a critical role in the decision point of cells to become rods or cones. In the context of the chicken retina, introduction of a dominant-negative Onecut1 (OC1-EnR) construct leads to a qualitative induction or both L-Maf and a reporter driven by the cow rhodopsin element [20]. This OC1-EnR dominant-negative construct encodes a fusion of the Engrailed (EnR) repressor domain to the DNA-binding domain of Onecut1 that is driven by the broadly active CAG promoter. To more quantitatively examine the formation of rod photoreceptors in response to OC1-EnR, E5 chicken retinas were electroporated with Rho::GFP reporter, Cag::Nucβ-gal, and either engrailed repressor alone (EnR) or the OC1-EnR fusion protein. Sections from retinas cultured for 3 days were examined by confocal microscopy for induction of GFP from the Rhodopsin reporter construct and also endogenous L-Maf protein expression, the earliest known marker of rods in the chicken. In retinas with introduced control EnR repressor construct, there were almost no Rho::GFP-positive cells or L-Maf positive cells in the electroporated population (Fig. 5a-d). In contrast, there was robust induction of the Rho reporter and L-Maf protein expression in retinas with introduced OC1-EnR (Fig. 5e-h). The majority of GFP-positive and L-Maf cells were located at the scleral surface of the retina where developing photoreceptors are found. These cells often had the appearance of photoreceptors with a short apical and basal process. There are also some GFP-positive cells located closer to the vitreal surface, though these were the minority of cells. Both of these populations appear to be formed in response to the OC1 dominant-negative construct and were examined for the presence of L-Maf. In the cells localized near the scleral surface, the majority of GFP-positive cells were also positive for L-Maf, congruent with the hypothesis that L-Maf is a major regulator of the rhodopsin reporter. The cells located closer to the vitreal surface did not typically express L-Maf, suggesting that either the GFP expression in these cells is independent of L-Maf regulation or L-Maf is not detectable in this cell population with the antibody that was used. Of all of the L-Maf positive cells that were electroporated, 82.7+/− 6.3% were Rho::GFP-positive, supporting the use of the cow Rho::GFP construct as a relatively good marker of rod photoreceptor cells. In addition, 58.2+/− 1.8% of the Rho::GFP positive cells expressed L-Maf. Given that the number of these induced cells is a small percentage of the electroporated population of cells (see below), this supports the use of this reporter as highly enriched activity in rod photoreceptors.

### Effects of Onecut1 dominant-negative on enhancer activity

We previously reported that introduction of Onecut1 dominant-negative constructs into the developing chicken retina led to upregulation of a rod cis-regulatory element activity and opposite effects on the ThrbCRM1 element [20]. To create a quantitative and efficient method to assess the activation of these reporters and to assess the effects of the dominant-negative on a wider range of cis-regulatory elements, a flow cytometry assay was developed. In this assay, retinas were co-electroporated with a UbiC::TdTomato construct, a GFP cis-regulatory element construct, and either the OC1-EnR or a control EnR only plasmid. After three days of culture, retinas were dissociated into single cells, fixed, and analyzed by flow cytometry.

The number of GFP positive cells relative to the number of TdTomato cells was calculated to determine the activity of the cis-regulatory element in the electroporated population. To determine if the Onecut1 dominant-negative had any effects on cis-regulatory activity, the percentages of cells with GFP expression were compared to retinas electroporated with a control engrailed repressor plasmid. GFP and TdTomato only cells were used to define a polygon gate (P6) in the double-positive quadrant that would only contain unambiguously double-positive cells (see Materials and Methods). We first tested the Rhodopsin::GFP element in this assay. In agreement with our previous report and the confocal data, the introduction of OC1 dominant-negative led to a robust induction of the rhodopsin promoter (207-fold), with extremely low levels of GFP in the absence of the dominant-negative construct (Fig. 5i, j, m). To determine if this effect could be an artifact of using the cow form of the rhodopsin promoter, we used a chicken rhodopsin reporter construct that was recently characterized and validated to preferentially drive expression in rhodopsin positive cells of the chicken retina [39]. As observed with the cow element, the chicken rhodopsin element drove minimal GFP reporter activity after three days of culture, but showed robust activity in the presence of the OC1-EnR dominant-negative (Additional file 8A, B). To determine whether the inductive effect on the chicken and cow rhodopsin elements was specific, a chicken red opsin GFP reporter construct was also tested [39]. In contrast to the rhodopsin reporters, there was a small repression of the red opsin construct in response to the introduction of OC1-EnR (Additional file 8C-F). Given that L-Maf is induced by OC1-EnR and the role of the related Nrl gene in directly regulating rhodopsin expression in mammals, it seemed plausible that OC1-EnR introduction led to induction of L-Maf, which then induced the activation of the rhodopsin reporter [12, 20]. In support of the functional equivalency of L-Maf for Nrl, it has recently been reported that chicken L-Maf can induce rod specific genes, including Rhodopsin, in the mouse retina [5]. To test this genetic pathway, the effects of additional L-Maf expression or a dominant-negative L-Maf construct on the induction of the Rhodopsin reporter by OC1-EnR was determined. A full-length L-Maf cDNA was placed under the control of the broadly active and robustly CAG element. When CAG::L-Maf was also introduced along with the rhodopsin reporter and CAG::OC1-EnR, there was an increased number of cells that expressed the Rhodopsin reporter, as well as an increase in the strength of the GFP reporter in the cells that were positive (Fig. 5m). To test if the induction of the Rhodopsin reporter by OC1-EnR is mediated by L-Maf, the induction assay was repeated with the inclusion of a dominant-negative L-Maf (L-Maf-EnR). Indeed, the co-introduction of CAG::L-Maf-EnR was able to significantly suppress the induction of the Rhodopsin reporter supporting a suggested role for L-Maf upstream of rhodopsin and downstream of Onecut1 (Fig. 5l, m). Misexpression of this wild-type form of L-Maf, without OC1EnR did induce the Rhodopsin reporter, though it was not significant compared to the EnR (Additional file 9). In contrast, the introduction of OC1EnR or co-electroporation with both L-Maf and OC1EnR led to a significantly greater induction than the EnR control (Additional file 9). The difference in activation of the Rhodopsin reporter between misexpression of OC1-EnR (through L-Maf induction) and L-Maf alone could be due to several reasons, including expression differences of the proteins or alterations in other genes besides L-Maf in response to the OC1-EnR that facilitate Rhodopsin reporter activation.

**Fig. 6:**
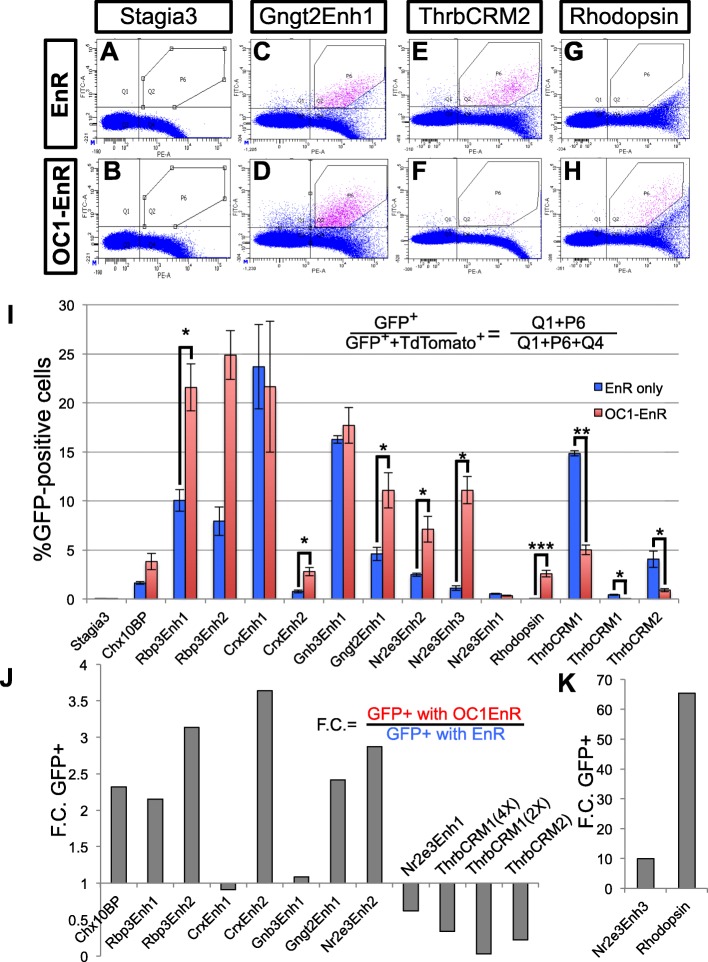
Effects of OC1-EnR on Photoreceptor CREs. **a**-**h** Flow cytometry examples of Stagia3 reporter constructs in response to OC1-EnR in the chicken retina. GFP reporter expression is measured along the y-axis and TdTomato (from UbiC::TdT) along the x-axis. The P6 gate marks unambiguous GFP, TdTomato double-positive cells. **i** Bar plot of the percentage of GFP-positive cells in the electroporated population for each Stagia3 reporter shown along the x-axis. Error bars represent S.E.M. from *N* > 3 biological replicates. Statistical analysis was performed with a student t-test or a Mann-Whitney t-test if tests of Normality (Shapiro-Wilk) or tests of Equality of Variances (Levene’s) were significant. *p*-values denoted as < 0.05(*). < 0.01(**). < 0.001(***). Only groups in which the displayed and replicate groups were significant are denoted as significant. **j** Fold change (F.C.) of the reporter noted along the x-axis calculated by dividing the OC1-EnR averages of GFP-positive cells by the average in response to the EnR control. **k** Fold change plot for the Nr2e3Enh3 and cow Rhodopsin elements as in J, but with an extended scale

To test more broadly the effects of the OC1-EnR construct on the gene regulatory networks during photoreceptor genesis, the effects on the regulatory elements described in Table 1 were tested in the flow cytometry assay (Fig. 6). Representative flow cytometry plots of four of these enhancers are shown (Fig. 6a-h). As expected, the Stagia3 plasmid without additional cis-regulatory elements had minimal GFP expression (less than 0.1% of cells in either the presence of EnR or OC1-EnR) (Fig. 6a, b). The Gngt2 element that was active in both the chicken cone and mouse rod assays was somewhat increased in response to the OC1-EnR compared to the EnR alone (Fig. 6c, d). In contrast, the cone-associated element ThrbCRM2 had decreased activity in response to OC1-EnR, while the rod-associated Rhodopsin element was increased (Fig. 6e-h). Quantitation of all of the elements tested under conditions of EnR alone or EnR-OC1 are shown in Fig. 6i. Several of these elements showed small but significant increases in activity when coelectroporated with OC1-EnR compared to EnR alone. This induction was up to 4 fold and was observed with all of the elements associated with both cone and rod activity in this studies’ previous assays (Rbp3, Crx, Gnb3, Gngt2) (Fig. 6i, j). This was also observed with the Chx10 bipolar construct that is not expected to be active in photoreceptors, suggesting that the OC1-EnR construct might have some mild non-specific activation or perhaps promotes some general retinal differentiation programs (Fig. 6i, j).

Two elements had a much larger OC1-EnR induction then the EnR control. One of these was the Rhodopsin element as shown previously in Fig. 4 and the other was the cNr2e3Enh3 element, with 65 fold and 10 fold changes respectively (Fig. 6i, k). There was also a set of elements that had a decrease in activity. One of these was the ThrbCRM1 element, which was previously reported to have a qualitative decrease in activity in response to the OC1-EnR element [20] (Fig. 6i, j). This effect was observed with two different forms of the element - one that had two copies of the ThrbCRM1 element and one that had 4 copies. This was expected given that this element is directly bound and activated by Onecut1 [20]. In addition, another element associated with the Thrb gene (ThrbCRM2) also showed a significant decrease in activity in response to OC1-EnR (Fig. 6i, j). Though this sequence has not been tested experimentally for binding to OC1, it does not have a predicted OC1 binding site and it is not active in RPCs where OC1 is predominantly expressed [20]. This suggests that the decrease in the activity of this element could be further down the gene regulatory cascade of Onecut1’s effects on cone development. The only other element that had a decrease in activity in response to OC1-Enr was the mNr2e3Enh1 element, which had very low activity in the chicken retina (Fig. 6i, j). This quantitative analysis suggests that Onecut1 is involved in specific photoreceptor gene regulatory networks and is consistent with a role in normally promoting cone-related networks and repressing rod photoreceptor networks.

### Cis-regulatory element activity in rods induced by the Onecut1 dominant-negative

**Fig. 7:**
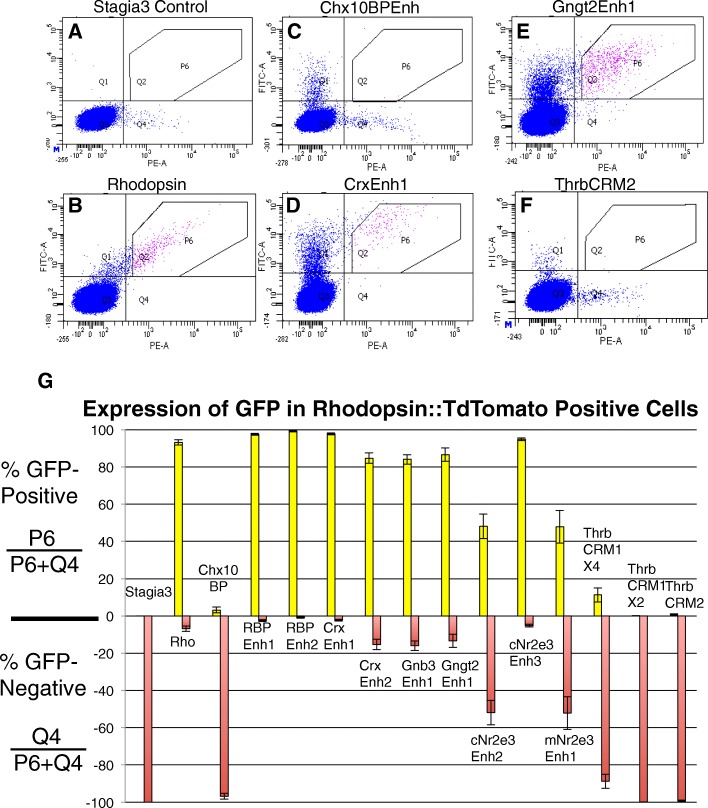
Activity of cis-regulatory elements in OC1-EnR induced rod photoreceptors. **a**-**f** Flow cytometry plots of dissociated chicken retinal cells coelectroporated with CAG::OC1-EnR, Rhodopsin::TdTomato, and cis-regulatory elements (CREs) driving GFP. Rhodopsin::TdTomato+, CRE::GFP+ are defined by the P6 gate and Rhodopsin::TdTomato+ cells are defined by the Q4 quadrant. **g** Plot of Rhodopsin::TdTomato+, CRE::GFP+/Total Rhodopsin::TdTomato+ cells in yellow bars and Rhodopsin::TdTomato+, CRE::GFP- /Total Rhodopsin::TdTomato+ cells in red bars. Note that the populations denoted by the yellow and red are derived from the same data and are both included for display purposes. Error bars represent S.E.M. from *N* > 3 biological replicates

The previous experiments characterized the activity of elements in chicken cones and mouse rods. We next wanted to test the rod activity of these elements in the context of the chicken retina and to determine whether the elements were downstream of Onecut1. To do so, an additional flow cytometry assay was developed. Retinas were co-electroporated with the OC1-EnR dominant negative construct, a Rho::TdTomato construct and one of the cis-regulatory constructs driving GFP. After three days of culture, retinas were dissociated and analyzed by flow cytometry for GFP and TdTomato fluorescence. The number of GFP-positive cells in the Rho::TdTomato-positive population were calculated. The effectiveness of this assay was determined using three controls. The first control was to use a Stagia3 control plasmid without cis-regulatory sequences. It was expected that most Rho::TdTomato-positive cells would be GFP-negative and this was indeed the case (0% GFP-positive) (Fig. 7a, g). The second control was to use a Rhodopsin::GFP construct, which should have the same regulation as the Rhodopsin::TdTomato construct. Indeed, retinas electroporated with this construct revealed that 93.2% of TdTomato-positive cells also express GFP (Fig. 7b, g). Lastly, a Chx10 bipolar regulatory element not expected to be active in rod photoreceptors based on its activity in the mouse retina was tested. These retinas were cultured for one week to allow for this cis-regulatory element to become active in the retina. As expected, only 3.2% of Rhodopsin::TdTomato positive cells were also GFP-positive. This was not due to a lack of GFP-positive cells as there were a large number of this single-positive population (Fig. 7c, g).

Each cis-regulatory element was tested individually in this Rhodopsin::TdTomato assay and the percentage of Tdtomato-positive cells was calculated and plotted (Fig. 7g). These cis-regulatory elements largely fell in to two groups - those with robust co-expression of the GFP reporter in the TdTomato population and those with little expression in the TdTomato population. Those that highly overlapped with Rhodopsin include the four Crx and Rbp3 elements, which would be expected if these elements were involved in the regulation of the known expression of these genes in both rod and cone photoreceptor types (Fig. 7d for CrxEnh1 example and Fig. 7g). Additionally, the newly identified Nr2e3Enh3 element was active in most rods, consistent with the expression of Nr2e3 in rods (Fig. 7g). As was observed for rods in the mouse retina, the Gnb3 and Gngt2 elements were also active in chicken rod cells induced by the Onecut1 dominant-negative (Fig. 7e, g). Given that the Gnb3 and Gngt2 genes are thought to be expressed in cones and not rods, this suggests that these elements are not recapitulating the normal regulation of their associated genes and is congruent with the observed activity of these elements in mouse rod photoreceptors (see Figs. 3 and 4).

Several elements were minimally active in rod photoreceptors. These included the Thrb elements that were tested. Both the ThrbCRM1 and ThrbCRM2 elements were also decreased in activity in response to the OC1-EnR (consistent with the UbiqC::TdT experiment), but the remaining GFP positive cells were found in the GFP only quadrant, suggesting that the lack of double positive cells was not because there was a complete lack of GFP (Fig. 7f, g). The only other elements that were not active in a majority of rods were the related Nr2e3Enh1 and Nr2e3Enh2 elements (Fig. 7g). These experiments further confirm that only the Thrb elements have specificity for cones and not rods.

### Activity of Nr2e3 Cis-regulatory elements in mouse retina

**Fig. 8:**
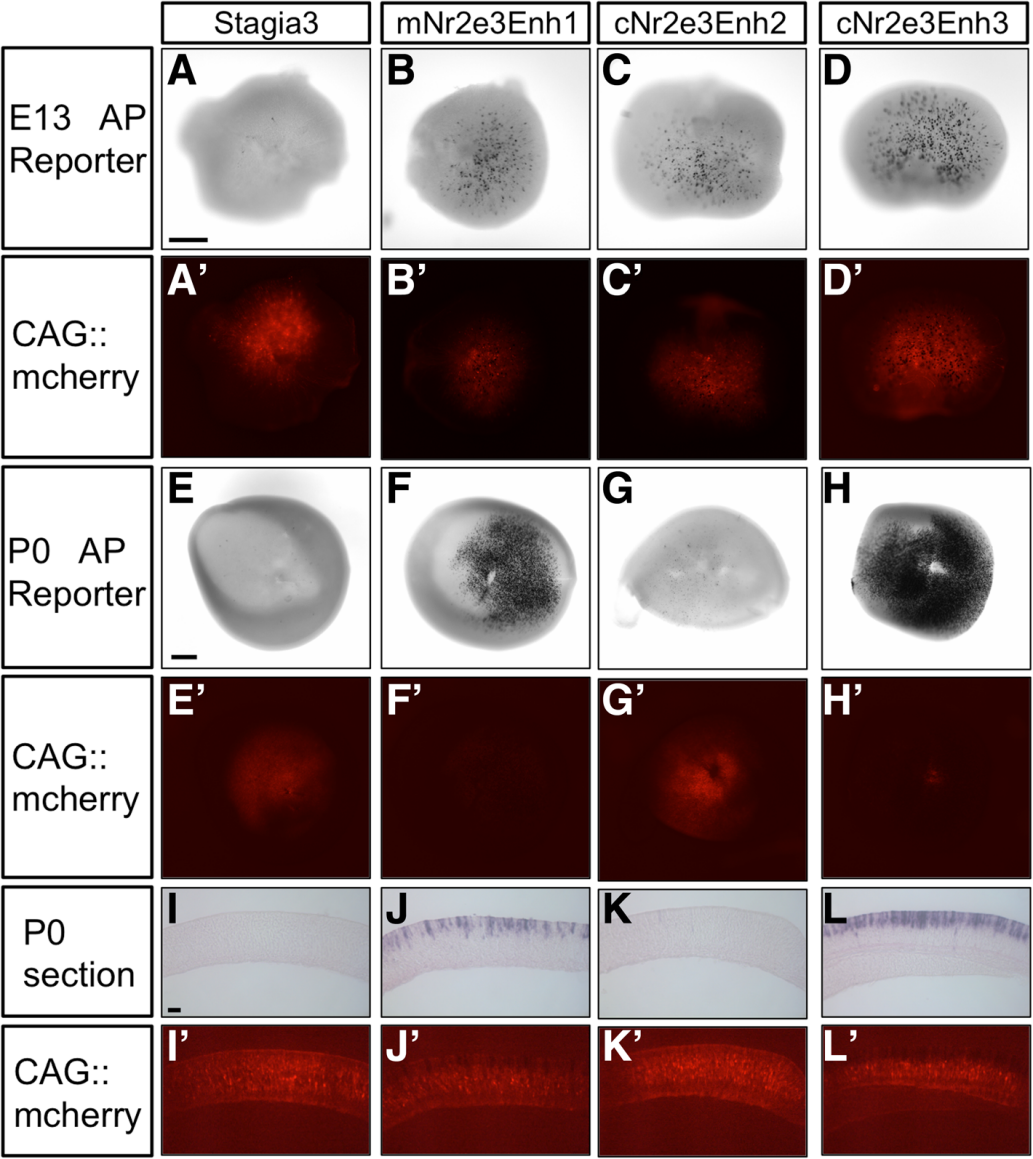
Activity of Nr2e3 cis-regulatory elements in mouse retinas. (A-D) Co-electroporation of one of the Nr2e3 elements driving an AP reporter or the control Stagia3 vector with CAG::mCherry (signal shown in A’-D’) to confirm electroporation in E13 mouse retinas. (E-H) Same DNA constructs as in A-D, but using P0 mouse retinas.(E’-H’) CAG::mCherry signal from retinas shown in E-H. (I-L) Sections of P0 retinas shown in E-H showing location of AP reporter-positive cells. (I’-L’) CAG::mCherry signal of sections shown in I-L. Scale bar in A is 100um and applies to A-D and A’-D’. Scale bar in E is 200 μm and applies to E-H and E’-H’. Scale bar in I is 20 μm and applies to I-L and I’-L’

The previous experiments, as well as a report in zebrafish, suggest that Nr2e3 is expressed transiently in cones, while it is persistently expressed in rods. In mice, Nr2e3 is generally considered a dedicated rod marker though it has been reported to be in cone photoreceptors during at least one timepoint of embryonic development [36, 43]. To determine whether any of the three characterized Nr2e3 elements were active in mouse cone photoreceptors, we analyzed both the activity of the Nr2e3 elements and the expression of endogenous Nr2e3 relative to the cone marker, Rxrg. We first determined if the three elements were active during the early embryonic timepoints when cone photoreceptors were being generated. We used a sensitive alkaline phosphatase (AP) assay in wholemount retinas to assess their activity. E13.5 retinas were electroporated ex vivo with reporter plasmids and a Cag::mCherry control, cultured for two days, and then processed for AP activity. Retinas electroporated with the control Stagia3 reporter plasmid had very few AP-positive cells. All three of the Nr2e3 elements showed robust activity in the E13 retina (Fig. 8A-D, A’-D’). The same DNA mixes were electroporated into P0 retinas, when only rod photoreceptors should be targeted by electroporation. These results qualitatively mirrored what was observed with GFP fluorescence (Fig. 8E-H, E’-H’, compare to Figs. 3 and 4). A small number of faint AP-positive cells were observed with the cNr2e3Enh1 element, which were not detectable with the GFP reporter. This could be due to the increased sensitivity of the AP reporter, or the timing of the assay, as this experiment occurred only 2 days after plasmid introduction. Sections of these retinas also confirmed these observations (Fig. 8I-L, I’-L’).

To more closely examine the activity of these elements in the embryonic mouse retina, we focused on the cNr2e3Enh2 and cNr2e3Enh3 elements. We first examined the distribution of GFP expression driven by these elements relative to co-electroporated ThrbCRM1::AU1 (active in RPCs that generate cones and cone photoreceptors) and CAG::Nucβ-gal (broad activity) [20]. While retinas electroporated with the Stagia3 control vector had little GFP expression in the AU1 or Nucβ-gal positive population, both of the Nr2e3 elements drove GFP expression primarily in the developing photoreceptor layer and co-localized with AU1 and Rxrg (Additional file 10).

**Fig. 9:**
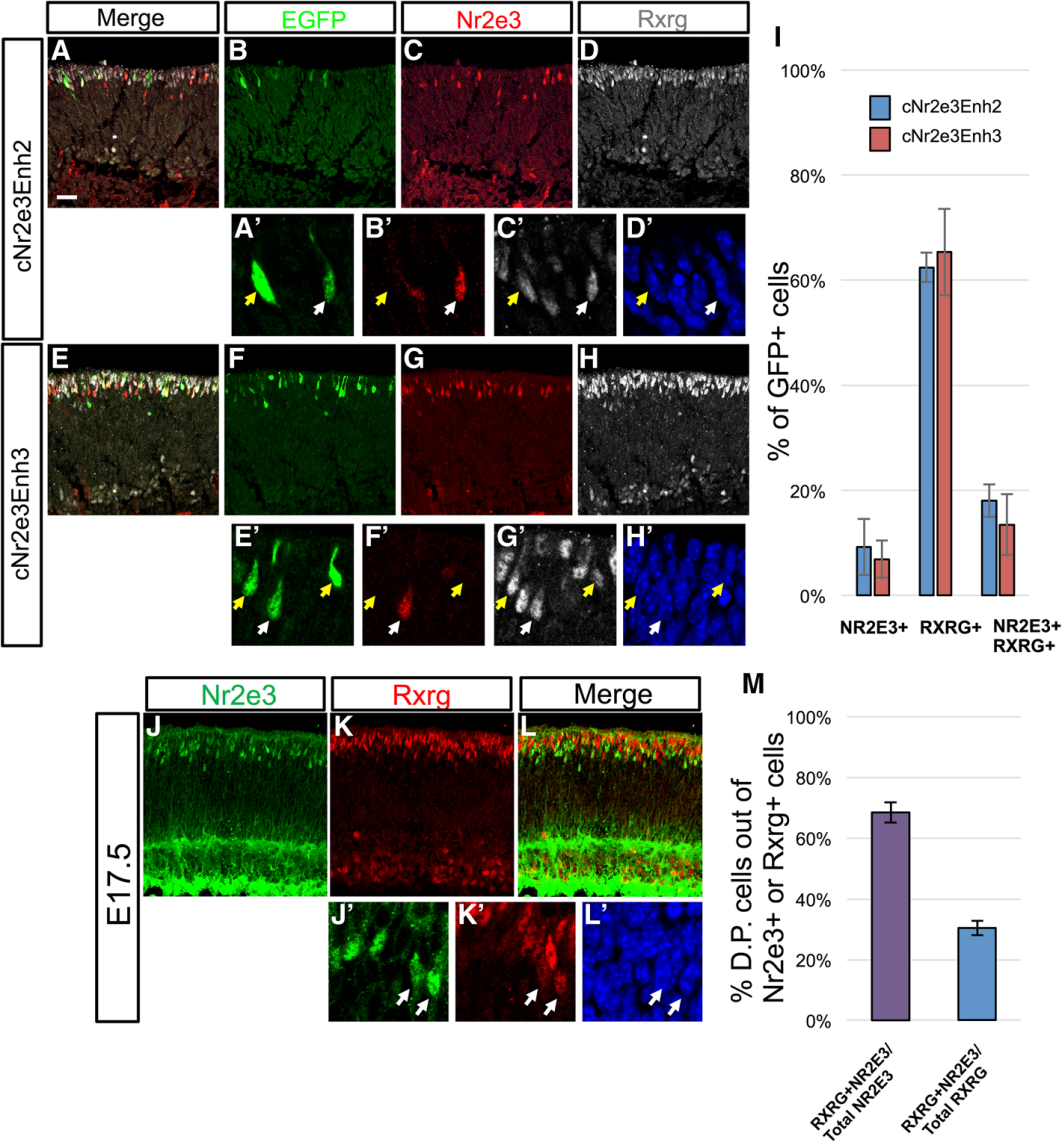
Coexpression of Rxrg and Nr2e3 in the embryonic mouse retina. (A-H) E14.5 mouse retinas electroporated with either the cNr2e3Enh2 or cNr2e3Enh3 plasmid, cultured ex vivo for 2 days, and processed for immunofluorescence confocal imaging of EGFP (green, chicken antibody), Nr2e3 (red, mouse antibody), Rxrg (white, rabbit antibody), and DAPI. Panels in A-H represent maximum projections of z-stacks with the depicted channel shown at the top of the column. The merge column has EGFP, Nr2e3, and Rxrg signals. (A’-H’) Magnified single z-plane images with the signals for (A’,E’) EGFP, (B’,F’) Nr2e3, (C’,G’) Rxrg, and (D’,H’) DAPI. White arrows point to GFP+ cells that also express Nr2e3 and Rxrg. Yellow arrows point to GFP+ cells that express Rxrg, but not Nr2e3. (I) A graph of the average percentage of GFP+ cells when driven by the cNr2e3Enh2 or cNr2e3Enh3 elements that express Nr2e3, Rxrg, or both Nr2e3 and Rxrg. (J-L) Maximum projection of a z-stack image of a E17.5 mouse retina processed for immunofluorescent detection of Nr2e3 (J, green) and Rxrg (K, red) or both (L, Merge). (J’-L’) Magnified single z-plane images of the same area visualized for signals for Nr2e3 (J’), Rxrg (K’), or DAPI (L’). (M) A graph of the average percentage of Nr2e3, Rxrg double-positive (D.P.) cells out of the total Nr2e3+ population (left bar) or the total Rxrg+ population (right bar). In both graphs *N* ≥ 3 biological replicates. Error bars represent standard error of the mean. All images are oriented with the scleral side of the retina at the top of the image. Scale bar in A represents 20 μm and applies to A-L.

To determine if the Nr2e3 elements were active in Nr2e3-positive cells and/or cone photoreceptors, embryonic mouse retinas were electroporated with one of the Nr2e3 GFP reporters, cultured ex vivo for 2 days and processed to detect endogenous Nr2e3 and Rxrg expression (Fig. 9). Within the GFP-positive populations, most of the cells expressed only Rxrg, with smaller populations of Nr2e3, Rxrg double-positive cells and Nr2e3 single-positive cells (Fig. 9A-I). This activity in Rxrg-positive cells suggests that these elements are indeed active in cone photoreceptors as was observed in the chicken retina. However, while these elements do drive in some Nr2e3-positive cells, the majority of cells do not express Nr2e3 (Fig. 9I). This could be due to several reasons, such as a transient expression of Nr2e3 protein, a lack of sensitivity with the Nr2e3 antibody, or that these elements only recapitulate a portion of endogenous Nr2e3 regulation.

The previous experiments determined that within the Nr2e3Enh2 and Nr2e3Enh3 populations, some cone photoreceptors marked by Rxrg do also express Nr2e3. To determine more broadly the co-expression of Rxrg and Nr2e3, wildtype E17.5 retinas were examined for these markers (Fig. 9J-M). Confocal imaging revealed cells positive for both transcription factors primarily in the developing photoreceptor portion of the retina. Additional signals were found in the inner retina with Rxrg found in some retinal ganglion cells at the vitreal side of the retina and a strong background signal in the Nr2e3 channel that is due to the use of a mouse monoclonal on mouse tissue (Fig. 9J-L). Quantification of cells in the upper half of the retina determined that the majority of Nr2e3-positive cells at this timepoint also express Rxrg (Fig. 9M). Examination of the Rxrg-positive population determined that approximately 30% of these cells also expressed Nr2e3 (Fig. 9M). This confirms that there are cone photoreceptors that express Nr2e3 during mouse embryonic development.

## Discussion

The generation of photoreceptors during development and the underlying gene regulation and differentiation processes has been a long-standing interest of the eye research community. One reason for this is that photoreceptors are a major target of retinal disease. The identification of new photoreceptor cis-regulatory elements active during development could be therapeutically useful in several ways. For instance, the activity of these elements can be used as to monitor the formation of early photoreceptors. This could be advantageous in that these elements could have more restricted activity than the genes that they control and so could provide more specific analytical tools. For instance, a Crx::GFP transgenic line is widely used to monitor the formation of photoreceptors, however, Crx is also expressed in bipolar cells and in other parts of the brain outside of the retina [44, 45]. The CrxEnh2 element or one of the other photoreceptor enhancers identified here could provide a more specific tool to identify newborn photoreceptors and not also bipolar cells. These elements could be used in viral vectors, transgenic animal models, or stem cells to allow for easy detection of specific gene regulatory events in these cells by having fluorescent reporters under their transcriptional control. In addition, they could be used to drive the expression of bioactive proteins, for instance, in a gene therapy approach. Elements with different transcriptional strength and specificity may be important depending on the gene to be delivered and/or the disease to be treated. This study examined both specificity and strength using the developing mouse and chicken retinas as models, to take particular advantage of the developmental timing of these species to target rods and cones, respectively. In addition, elements were isolated primarily from mouse and chicken genomes, which may affect the activity of the element given the species of origin and the associated evolutionary pressures exerted on that species (see below discussion of the Nr2e3 elements). Whether functional changes exist between species variants of any of the elements tested here has not yet been determined, but could be of interest in the context of the evolution of visual system, or for therapeutic reasons, to identify elements that have changes that could be beneficial in gene therapy approaches.

The molecular and cellular events that occur during the genesis of rod and cone photoreceptors during development are not well understood. One proposed model is that a postmitotic photoreceptor precursor is generated during development and this cell follows a rod fate if it begins to express Nrl and a cone fate if it does not [19]. Recent studies have uncovered a function for the Onecut1 and 2 transcription factors in repressing the rod fate upstream of Nrl [20, 21]. In the current study, we used a quantitative assay to characterize this effect using a Rhodopsin reporter as an output. Effects of modulating L-Maf expression in these experiments supports the model that Onecut1 acts upstream of L-Maf to repress the rod fate. The expression of the Onecut factors in RPCs suggests that the critical gene regulatory event for the activity of the Nrl switch could be initiated in these dividing cells instead of the postmitotic photoreceptor [20, 21]. These Onecut factors have also been found to positively regulate early cone genes, such as Thrb, through the ThrbCRM1 element, and Rxrg, suggesting they promote at least some aspects of the cone gene regulatory network though the extent of this effect has not yet been fully elucidated [20].

**Fig. 10:**
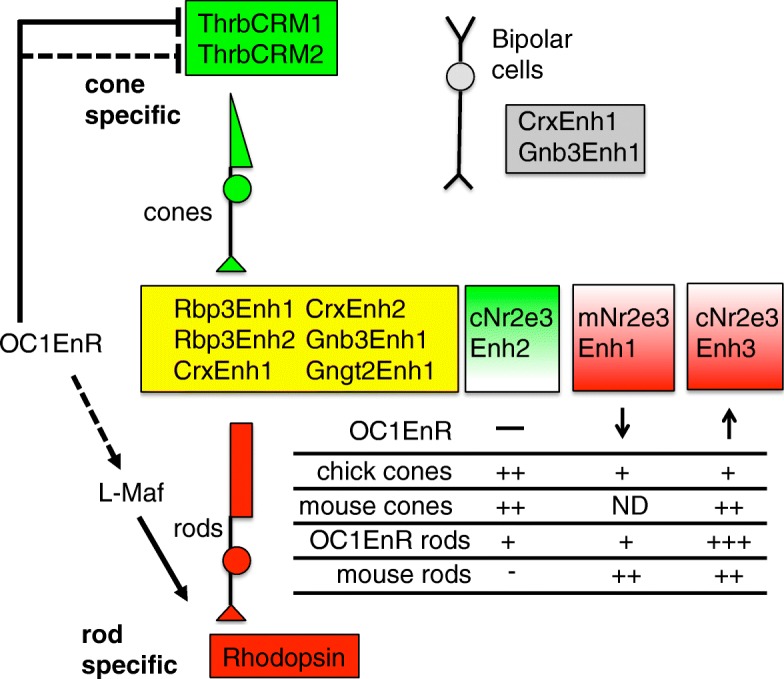
Summary of the activity of cis-regulatory elements tested in this study. The elements shown in yellow are active in both cone and rod photoreceptors in a reporter assay. The CrxEnh1 and Gnb3Enh1 elements are also active in developing bipolar cells. The ThrbCRM1 and ThrbCRM2 elements (in green shading) are cone specific and the Rhodopsin element is rod specific in the assays used in this study. The Nr2e3 elements show a less clear segregation to cone or rod specific activity as illustrated in the summary of the assays shown below. ND is Not Determined. Dashed lines represent putative indirect effects

The effects of inhibiting the OC1 regulatory network was extended in experiments described here through examination of other regulatory elements in addition to the ThrbCRM1 element (Fig. 10). All of these elements are found in close proximity to a gene of interest and are likely to participate in the regulation of the corresponding gene, though this has not been definitively demonstrated. While the cone and/or rod activity of some enhancer elements matched what was known about the expression of their corresponding gene, two notable exceptions were identified. The first was that elements associated with two cone genes, Gnb3 and Gngt2, were active in both cones and rods. This suggests that these particular elements are activated by the same gene regulatory network in both cell types or they respond to cell type specific networks of both cones and rods. If, in fact, these genes are not normally transcribed in rods, this suggests that there are other elements or mechanisms that normally act to generate this transcriptional specificity. The second exception was that the activity of elements associated with the Nr2e3 gene were also active in both rods and cones. Most models of Nr2e3 expression place it as a dedicated rod marker that is expressed downstream of Nrl in rod photoreceptors. However, while this restricted expression has been observed in adult photoreceptors, two previous studies have suggested that Nr2e3 is in fact expressed in cone photoreceptors during development in both zebrafish and mice [16, 36]. We show here that the Nr2e3 gene is also expressed in the chicken retina prior to the earliest known chicken marker of rods, L-Maf, suggesting that Nr2e3 is also expressed in cone photoreceptors in this species. In addition, two of the Nr2e3 elements drove reporter activity in embryonic mouse cone photoreceptors and endogenous Rxrg and Nr2e3 were found to be co-expressed in these reporter labeled cells and in wildtype retinas. Though the elements proximal to Nr2e3 used in this study show activity in both chick and mouse rods and cones, it is interesting to note that there were quantitative differences in this activity. The novel Nr2e3Enh3 identified in this study shows a robust activation in mouse rods similar to the mouse Enh1 that was previously identified. In addition, this cis-regulatory region increased its activity substantially more than any other element except for Rhodopsin in response to introduction of Oc1-EnR. This element also appears to be active in cone photoreceptors of both chicks and mice. For mNr2e3Enh1 and cNr2e3Enh2, these similar, and perhaps homologous, elements behaved quite differently in the context of the developing chicken and mouse retinas. Overall, the chicken form had higher activity in cone photoreceptors and the mouse form in rod photoreceptors, though the mouse form did not respond to OC1EnR expression as the cNr2e3Enh3 or the rhodopsin constructs did. Whether the differences between these elements, were due to changes in transcription factor binding sequences, or some other sequence differences that are less specific has not yet been determined. One possibility is that these differences could be relevant to the species-specific adaptation of mouse and chicken retinas to a nocturnal or diurnal lifestyle, respectively. Experiments in the adult mouse retina have shown the dramatic loss of Nr2e3 expression in Nrl mutants in the postnatal retina suggesting that Nrl is a major driver of Nr2e3 transcription. Presumably, other transcription factors are responsible for Nr2e3 transcription in cone photoreceptors given the lack of Nrl or L-Maf expression in these cells. Further experiments will be necessary to define the gene regulatory network that activates Nr2e3 in cones and whether it has any function in these cells. In addition, the temporal and spatial (for instance along the dorsal-ventral axis) parameters of expression of Nr2e3 in cones of both the chicken and mouse needs to be further delineated and correlated to the elements described here.

The study of dynamic cis-regulatory events during development is difficult due to multiple issues. One is the lack of quantitative measures of activity, especially with regard to particular cell types. In this study, quantitation was used in two ways. One was to determine the number of cells and type of cells with enhancer activity in the context of a developing retina using confocal microscopy and manual counting. These same enhancers were quantitatively measured for overall activity in the chicken retina and also in response to the OC1 dominant-negative construct using flow cytometry assays. This has the advantage of providing a robust and high-throughput platform to analyze new cis-regulatory elements in early cone and rod photoreceptors. Examination of several elements also reveals differences in response to Onecut1. As expected, the elements associated with the cone gene Thrb were repressed in response to OC1-EnR. Of those elements that were not repressed, there were essentially three groups. Most elements were induced between 2 and 4 fold, and the fact that the bipolar specific Chx10BP element was also induced to this level, suggests that this could be a non-specific effect of the OC1-EnR protein. The Rhodopsin elements and Nr2e3Enh3 elements were induced to a much greater extent, suggesting that they are highly enriched in rods compared to the other elements. The two elements that were not induced at all relative to EnR were CrxEnh1 and Gnb3Enh1. It is intriguing that these two elements are the two elements that also show bipolar expression in the mouse retina. Their differential response compared to those that are active in both cones and rods could signify that they are under the control of distinct gene-regulatory networks. Further studies that identify the gene regulatory networks that function downstream of Onecut1 will inform our understanding of the molecular events that underlie cone and rod photoreceptor formation.

## Conclusions

The gene regulatory networks that are active during the formation of rod and cone photoreceptors are likely to include those active in both cell types, in one of the photoreceptor classes, or in photoreceptors and other cell types, such as the related bipolar cell. This analysis quantitatively demonstrates clear differences between cis-regulatory elements in terms of this cellular specificity, and identifies new cis-regulatory elements that could be useful in gene therapy or cell identification. In addition, targeted evaluation of the role of Onecut1 in these gene regulatory networks was evaluated by high-throughput flow cytometry assays and suggests that Onecut1 does not play a general role in photoreceptor development but has a specific function in gene regulatory networks involved in promoting aspects of cone genesis and repressing those of rod photoreceptors. This study also identifies and characterizes the activity of cis-regulatory elements associated with the Nr2e3 gene. This analysis suggests that these Nr2e3 elements are active in cone development and have divergent activity in rods and relation to the Onecut1 gene regulatory network.

## Additional files

Additional file 1: **Table S1.** Table of cis-regulatory elements used in this study. The predominant expression by photoreceptor type of the associated gene is shown in the first column. The associated gene name is shown in the second column. The element name is shown in the third column and the genomic coordinates are shown in the 4th column. (XLSX 32 kb)

Additional file 2: **Table S2.** Table of primers used in this study. The purpose, primer name, and primer sequence in 5' to 3' orientation are listed. (XLSX 41 kb)

Additional file 3: Sequence similarity of CrxEnh1 and CrxEnh2 and Nrl occupancy. A) Sequence Lineup of CrxEnh1 Homologs from a Subset of Mammals and the CrxEnh2 Element. ClustalW-generated sequence alignment of 5 selected mammals shown to the left. Asterisks are shown below nucleotide positions that are conserved in all 5 species. A portion of the mCrxEnh2 sequence that is similar to the conserved portion of CrxEnh1 is shown below. Yellow shading identifies sequences conserved between the mouse CrxEnh1 and the mouse CrxEnh2 elements. B) Nrl occupancy of the CrxEnh1 and CrxEnh2 elements by Nrl protein. Top track depicts bigwig representation of sequences immunoprecipitated by Nrl antibodies and bottom track those immunoprecipitated by an IgG control (adopted from Hao et al. [33]. (PDF 370 kb)

Additional file 4: Activity of GFP reporters in cell types other than photoreceptors in the chicken retina. Retinas electroporated with a Cag::Nucβ-gal and the GFP reporter shown to the left of panels and imaged by confocal microscopy for the expression of GFP (green, rabbit antibody), Nucβ-gal (orange, chicken antibody), and Pax6 (purple, mouse antibody). The scleral portion of the retina is located near the top of the image. Scale bar in top left panel represents 20 μm and applies to all panels. (PNG 9638 kb)

Additional file 5: Chicken Nr2e3 RNA in situ hybridization on E6 chicken retinas. Scleral side of the retina is positioned at the top of the picture. Scale bar represents 20 μm. (PDF 298 kb)

Additional file 6: Activity of Nr2e3 Reporters in Rxrg-positive cone photoreceptor. E5 chicken retinas electroporated with Nr2e3Enh::GFP plasmids, cultured for 2 days ex vivo and processed for immunofluorescence detection of DAPI (blue), Nucβ-gal (red, chicken antibody), EGFP (green, rabbit antibody), and Rxrg (white, mouse antibody). (A-H) Maximum projections of confocal z-stacks with the channels shown above each column. (A’-H’) High magnification, single z-planes of the images shown in A-H. Arrows point to GFP, Rxrg double-positive cells and are in the same location in each image of each row. Scale bar in A applies to all panels and represents 20 μm in A-H and 4 μm in A’-H’. Retina is oriented with scleral surface at the top of each image. (PNG 9506 kb)

Additional file 7: Electroporation of mouse postnatal day 0 retinas does not efficiently target cone photoreceptors. (A-H) Mouse P0 retinas electroporated with CAG::GFP and Rbp3Enh1::GFP, cultured ex vivo for 8 days, and processed for immunofluorescence confocal imaging to detect EGFP (green, chicken antibody), Rxrg (red, mouse antibody), Cone Arrestin (white), and DAPI (blue). (A-D) Maximum projection of a z-stack showing the (A) EGFP, Rxrg, Cone Arrestin merged signals, (B) EGFP, (C) Rxrg, and (D) Cone arrestin (E-H) Single z-plane showing signals for (E) EGFP, (F) Rxrg,(G) Cone Arrestin, (H) DAPI (I) Bar graph displaying percentage of electroporated ONL cells (cells with GFP signal driven by CAG and/or Rbp3Enh1) with Rxrg immunoreactivity. *N* = 3 biological replicates. Error bar represents standard error of the mean. Scale bar in A represents 20 μm and applies to A-D. (PDF 4854 kb)

Additional file 8: Response of chicken Rhodopsin and Red Opsin elements to a OC1-EnR dominant negative construct. (A-D) Retinas were electroporated with the UbiC::TdTomato co-electroporation control, the Rhodopsin or Red opsin GFP reporter shown along the y-axis and the EnR construct shown at the top of each plot. E) Quantification of GFP-positive cells in the electroporated population. Bars represent averages of 4 biological replicates and error bars represent S.E.M. F) Fold change (F.C.) of the reporter noted along the x-axis calculated by dividing the OC1-EnR averages of GFP-positive cells by the average in response to the EnR control. (PDF 299 kb)

Additional file 9: Induction of Rhodopsin::GFP reporter by OC1-EnR and L-Maf. Retinas were electroporated with UbiC::TdTomato, cow Rhodopsin::GFP, and a construct that encodes the protein driven by CAG noted on the x-axis. The percentage of electroporated cells (marked by UbiC::TdT) that activate the cow Rhodopsin reporter is plotted along the y-axis. Error bars represent standard error of the mean. Statistical analysis using Dunnett’s test for comparison of L-Maf group to 3 experimental groups. Significance value denoted by * = 0.01, ** = < 0.001 and N.S. signifies “No Significance”. *N* = 4 biological replicates for each condition. (PDF 147 kb)

Additional file 10: Distribution of cells with Nr2e3Enh2 and Nr2e3Enh3 activity in the embryonic mouse retina. Embryonic day 14.5 retinas were co-electroporated with the Stagia3 plasmid shown to the left, ThrbCRM1::AU1, and CAG::Nucβ-gal, cultured for 2 days and processed for immunofluorescence confocal imaging for EGFP, AU1, β-gal, and DAPI. Maximum projections of z-stacks are shown in panels A-L with the channel depicted at the top of the column. Merge contains signals from EGFP, AU1, and β-gal channels. Single z-planes are shown below in the order of EGFP, AU1, β-gal, and DAPI from left to right. Arrows point to the position of ThrbCRM1::AU1+, Rxrg+ cells in each picture. In each image, the retina is oriented with the scleral portion at the top. Scale bar in A is 20 μm and applies to panels A-L. (PNG 9369 kb)

## Abbreviations

E5: Embryonic day 5
ECR: Evolutionary conserved regions
EnR: Engrailed repressor
GFP: Green fluorescent protein
INL: Inner nuclear layer
ONL: Outer nuclear layer
P0: Postnatal day 0
PCR: Polymerase chain reaction
RPC: Retinal progenitor cell
Stagia3: Stop Tata GFP ires AP version 3
UCSC: University of California Santa Cruz
