# Supplementary material for: Identification and characterization of early photoreceptor cis-regulatory elements and their relation to Onecut1
Source: Neural Dev. 2018 Nov 22;13:26. doi: 10.1186/s13064-018-0121-x (PMC6251108; doi:10.1186/s13064-018-0121-x)

Additional file 3

A

```
Dolphin       CTCTCTCCTTACCCCCATACGTCTCTGACCTCCCTCATGTTCCT-CCTGATCTCTGAGCC
Armadillo     CCTGCACTTTACACCGACA-GTCTCTGCCCCGCCC---GTTCCT-CCTGGAGCCGCAGCC
Baboon        CTCTTCCCTCACCCCCATATGTCTCTGTCCCTCCT---GGTCTC-CAAACACCCTC--CC
GuineaPig     CCCCCTTTCCACCTCCCCT-GTCCCTGATG-GCTGGACACCCTC-TCCCCTCCCCACCCC
Mouse         --CTTCCATTATTTGCATTTTTGTCTTCACCACTGTAGCTTATTGTTTCCCCCTTCCACC
                        *          *  **      *                         **

Dolphin       CCTCCCTGGATGTTTCCTCTGTCCCTGAACAGGACTAATTTAGCAAATGCTCCAATCCTC
Armadillo     CCTGCCTGACGGCCTCCTCTGGCCCTGACCAGGCCTAATTTAGCAAATGCTCCGATCCGC
Baboon        TTCCCCCAGCTGTTTCCTCTGCCCCTGACCAGGACTAATTTAGCAAATGCTGCAATCCTC
GuineaPig     CATCCCAGGCTGTTTTCTCTGTCCCTGCCTAGGACCAATTTAGCAAATTCTCCAATCCTC
Mouse         CCAGGCATTCTGTTTCCTGTGTCCCTGGACAGGACTAATTTAGTAAATGCTCCAATCCTC
                   *     *  * ** ** *****   *** * ******* **** ** * **** *
                                        mCrxEnh2  ..TTTAGCAAATACT-CAATCCTC

Dolphin       TTATG---ATGGTCCAGCCTCAGCAGAATGGCAGGAGAATTAGCGGGGATTAGACACAGA
Armadillo     TTA-G---CCGGTGCAGCCTCAGCAGAAGGGCGGGG--ATTAGCGGGGCTTAGGGACA--
Baboon        TTATGGCAACAGCCCAGCCTCAGCAGAATGGCAGGGGAATTAGCGAGGATTAGGTTCAGA
GuineaPig     TTAGGACGACTGTCCAGCCTCAGCAGAATGGCAGGG-AATTAGGGAGGATTACGTTCAGA
Mouse         TTAGGACAACAGTCCAGCCTCAGCAGAAAGGCAGGGGAATTATGGAGGATTAGGCTCAGC
              *** *      *  ************** *** **   ****  * ** ***    **
mCrxEnh2      TTAAG----CCACATGGCCTCAGCA..

Dolphin       CGCATGACCCATGGGGGAAAGGTACTTTGTGGAGACA-ACTGAATTTTAACTAAGTGAT-
Armadillo     CTCGTGACCCTCGCGGCA--GGCACTGCATGGAGACA-GCGGACATTGAGCTATCCCCCC
Baboon        CGCATGACCCGTGGGGGAG-GGGACTTCGGGGAGTTTTATCGAAATGATGCCCCCTCCTC
GuineaPig     CTCG-AGCCTGTGGGGGA--GGGGCTTCACAGAGACA-ACAGCTGTTACCCGGTGGTGG-
Mouse         CTCAGGACTTGTGGGGGAG-GGGACTCTGTAGGGACA-GCCATTTTCTTTTTAAACTAT-
              * *    *    * ** *  **  **     * *           *
```

B

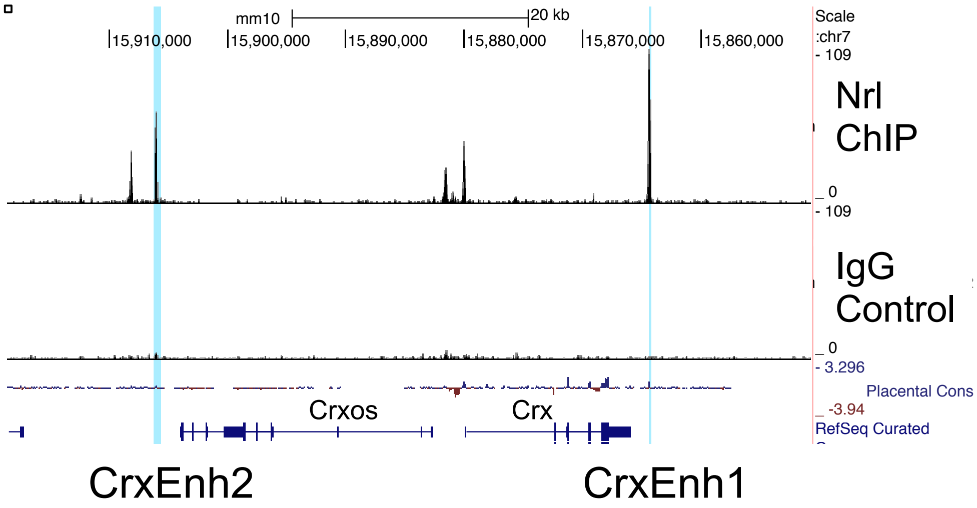

Supplement: Supplementary file 3 — Sequence similarity of CrxEnh1 and CrxEnh2 and Nrl occupancy. A) Sequence Lineup of CrxEnh1 Homologs from a Subset of Mammals and the CrxEnh2 Element. ClustalW-generated sequence alignment of 5 selected mammals shown to the left. Asterisks are shown below nucleotide positions that are conserved in all 5 species. A portion of the mCrxEnh2 sequence that is similar to the conserved portion of CrxEnh1 is shown below. Yellow shading identifies sequences conserved between the mouse CrxEnh1 and the mouse CrxEnh2 elements. B) Nrl occupancy of the CrxEnh1 and CrxEnh2 elements by Nrl protein. Top track depicts bigwig representation of sequences immunoprecipitated by Nrl antibodies and bottom track those immunoprecipitated by an IgG control (adopted from Hao et al. [33]. (PDF 370 kb) [file 13064_2018_121_MOESM3_ESM.pdf]
