# Supplementary figures and images for: Identification and characterization of early photoreceptor cis-regulatory elements and their relation to Onecut1
Source: Neural Dev. 2018 Nov 22;13:26. doi: 10.1186/s13064-018-0121-x (PMC6251108; doi:10.1186/s13064-018-0121-x)

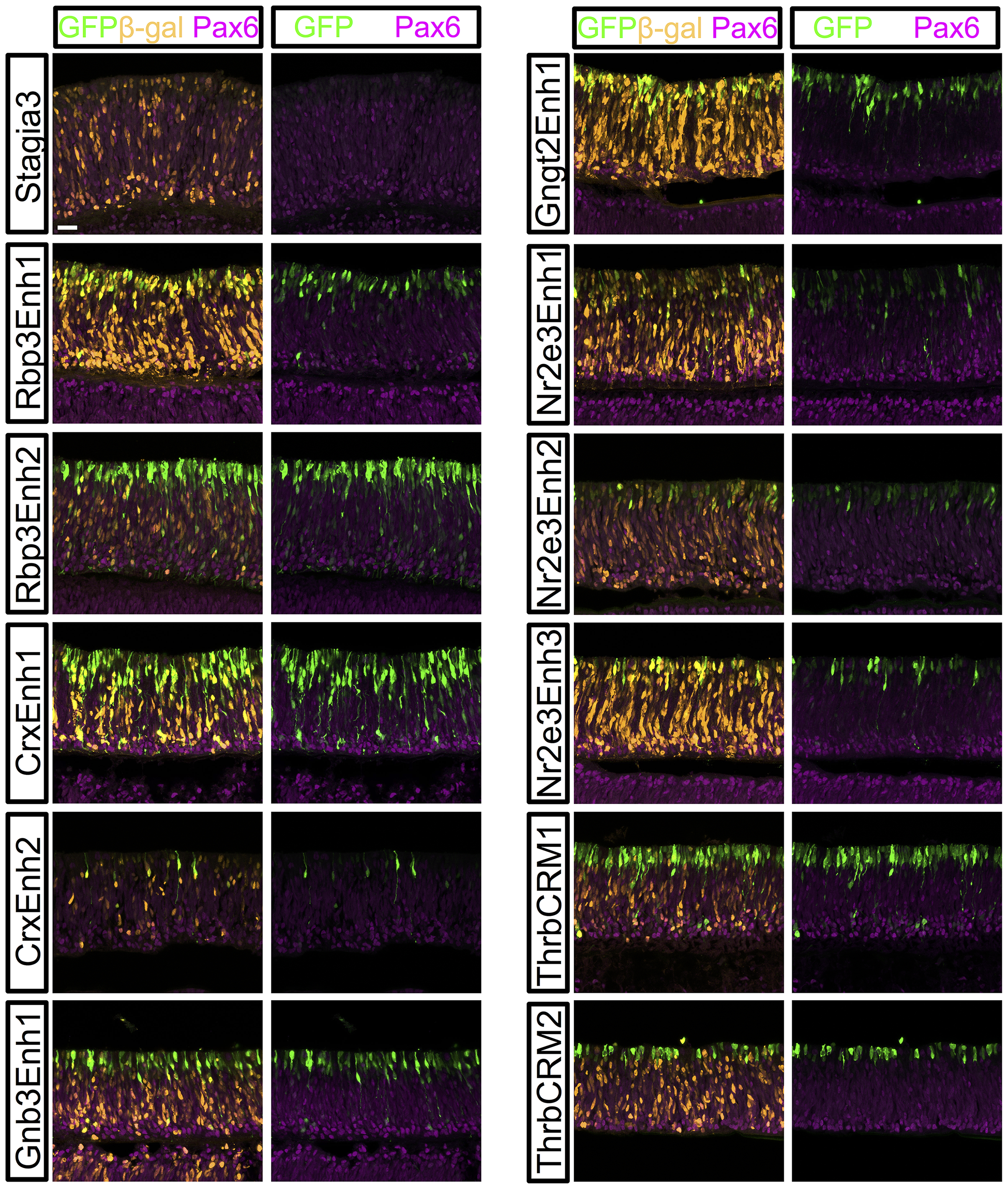

Supplement: Supplementary file 4 — Activity of GFP reporters in cell types other than photoreceptors in the chicken retina. Retinas electroporated with a Cag::Nucβ-gal and the GFP reporter shown to the left of panels and imaged by confocal microscopy for the expression of GFP (green, rabbit antibody), Nucβ-gal (orange, chicken antibody), and Pax6 (purple, mouse antibody). The scleral portion of the retina is located near the top of the image. Scale bar in top left panel represents 20 μm and applies to all panels. (PNG 9638 kb) [file 13064_2018_121_MOESM4_ESM.png]

## Additional file 5

Nr2e3 mRNA

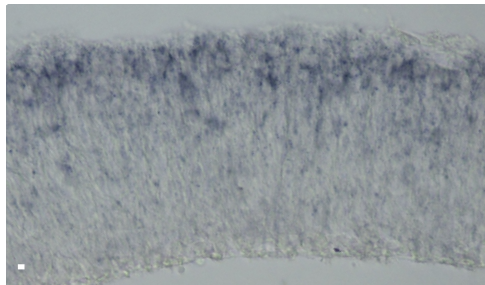

Supplement: Supplementary file 5 — Chicken Nr2e3 RNA in situ hybridization on E6 chicken retinas. Scleral side of the retina is positioned at the top of the picture. Scale bar represents 20 μm. (PDF 298 kb) [file 13064_2018_121_MOESM5_ESM.pdf]

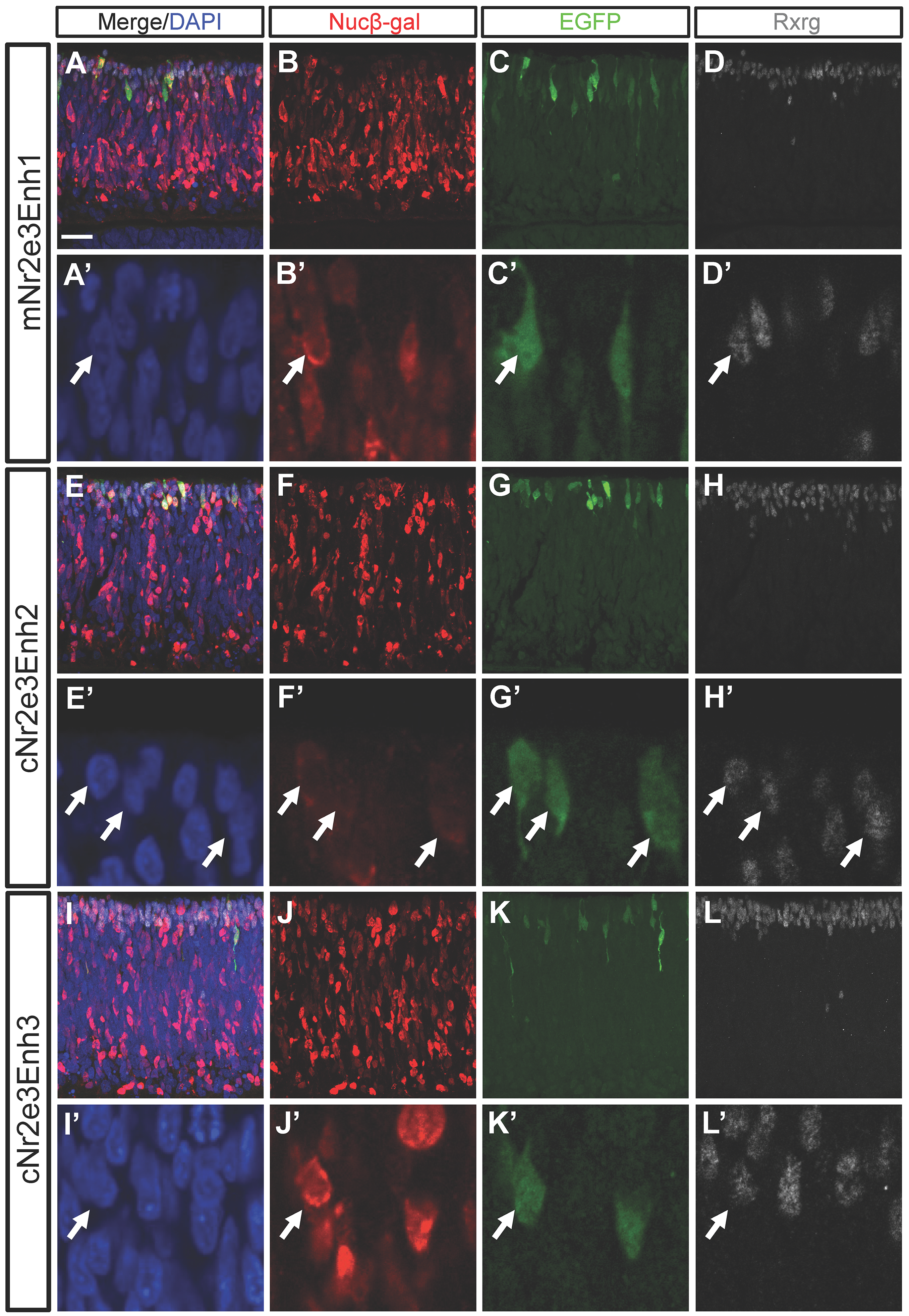

Supplement: Supplementary file 6 — Activity of Nr2e3 Reporters in Rxrg-positive cone photoreceptor. E5 chicken retinas electroporated with Nr2e3Enh::GFP plasmids, cultured for 2 days ex vivo and processed for immunofluorescence detection of DAPI (blue), Nucβ-gal (red, chicken antibody), EGFP (green, rabbit antibody), and Rxrg (white, mouse antibody). (A-H) Maximum projections of confocal z-stacks with the channels shown above each column. (A’-H’) High magnification, single z-planes of the images shown in A-H. Arrows point to GFP, Rxrg double-positive cells and are in the same location in each image of each row. Scale bar in A applies to all panels and represents 20 μm in A-H and 4 μm in A’-H’. Retina is oriented with scleral surface at the top of each image. (PNG 9506 kb) [file 13064_2018_121_MOESM6_ESM.png]

Additional file 7

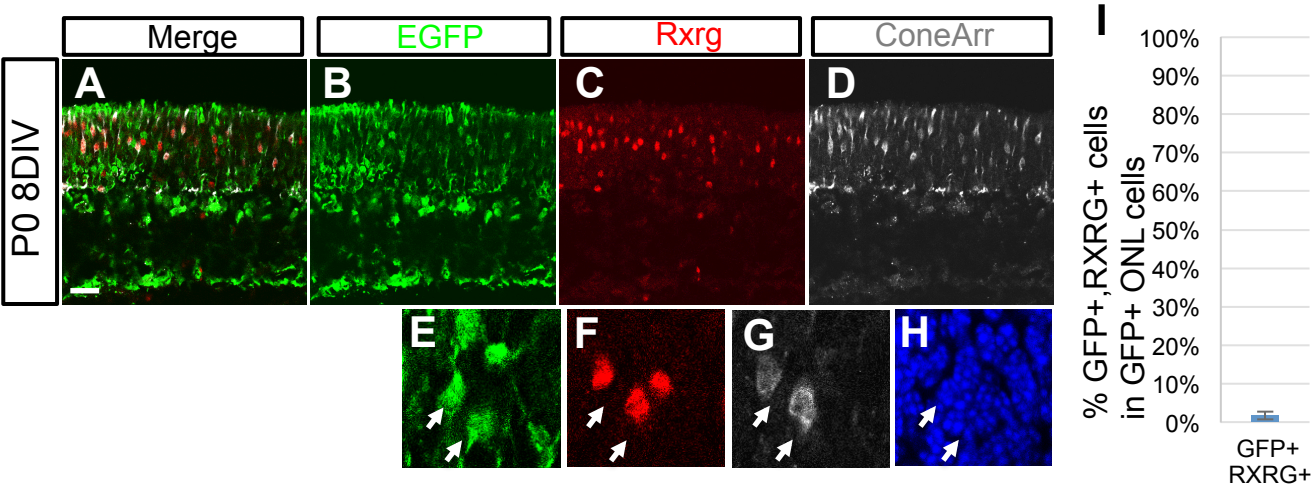

Supplement: Supplementary file 7 — Electroporation of mouse postnatal day 0 retinas does not efficiently target cone photoreceptors. (A-H) Mouse P0 retinas electroporated with CAG::GFP and Rbp3Enh1::GFP, cultured ex vivo for 8 days, and processed for immunofluorescence confocal imaging to detect EGFP (green, chicken antibody), Rxrg (red, mouse antibody), Cone Arrestin (white), and DAPI (blue). (A-D) Maximum projection of a z-stack showing the (A) EGFP, Rxrg, Cone Arrestin merged signals, (B) EGFP, (C) Rxrg, and (D) Cone arrestin (E-H) Single z-plane showing signals for (E) EGFP, (F) Rxrg,(G) Cone Arrestin, (H) DAPI (I) Bar graph displaying percentage of electroporated ONL cells (cells with GFP signal driven by CAG and/or Rbp3Enh1) with Rxrg immunoreactivity. N = 3 biological replicates. Error bar represents standard error of the mean. Scale bar in A represents 20 μm and applies to A-D. (PDF 4854 kb) [file 13064_2018_121_MOESM7_ESM.pdf]

# Additional file 8

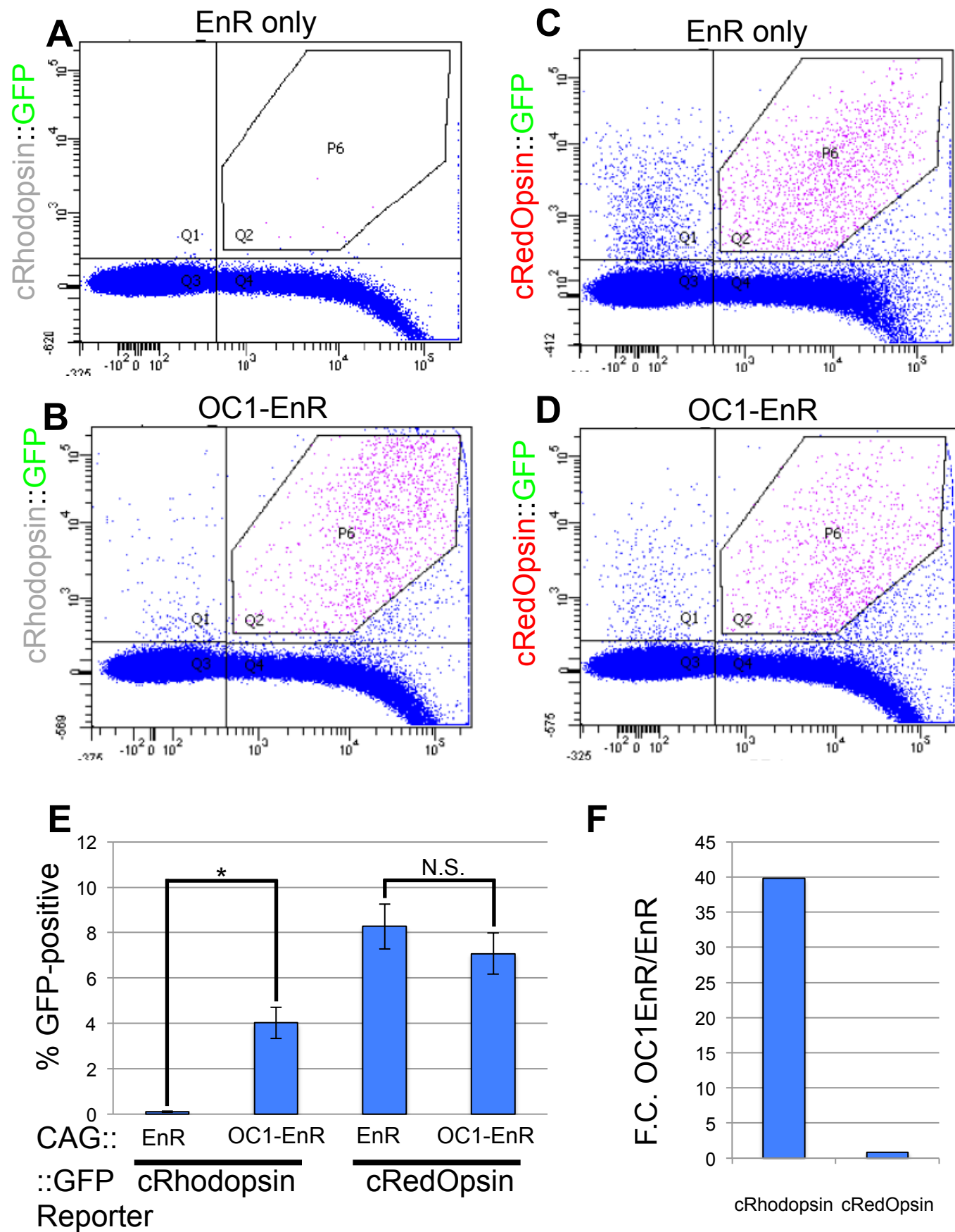

Supplement: Supplementary file 8 — Response of chicken Rhodopsin and Red Opsin elements to a OC1-EnR dominant negative construct. (A-D) Retinas were electroporated with the UbiC::TdTomato co-electroporation control, the Rhodopsin or Red opsin GFP reporter shown along the y-axis and the EnR construct shown at the top of each plot. E) Quantification of GFP-positive cells in the electroporated population. Bars represent averages of 4 biological replicates and error bars represent S.E.M. F) Fold change (F.C.) of the reporter noted along the x-axis calculated by dividing the OC1-EnR averages of GFP-positive cells by the average in response to the EnR control. (PDF 299 kb) [file 13064_2018_121_MOESM8_ESM.pdf]

## Additional file 9

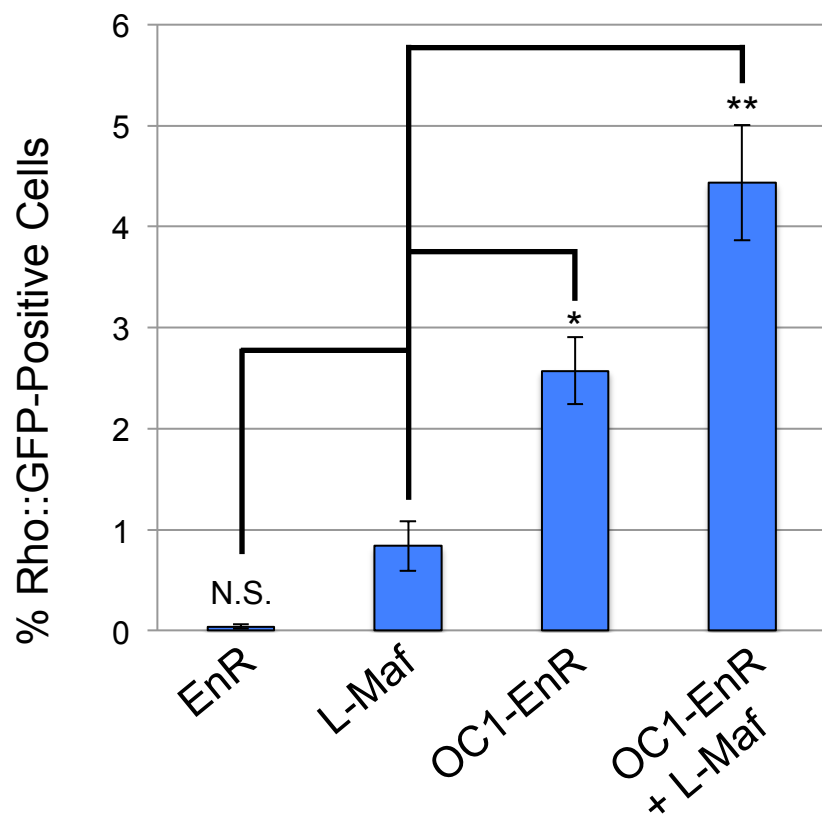

Supplement: Supplementary file 9 — Induction of Rhodopsin::GFP reporter by OC1-EnR and L-Maf. Retinas were electroporated with UbiC::TdTomato, cow Rhodopsin::GFP, and a construct that encodes the protein driven by CAG noted on the x-axis. The percentage of electroporated cells (marked by UbiC::TdT) that activate the cow Rhodopsin reporter is plotted along the y-axis. Error bars represent standard error of the mean. Statistical analysis using Dunnett’s test for comparison of L-Maf group to 3 experimental groups. Significance value denoted by * = 0.01, ** = < 0.001 and N.S. signifies “No Significance”. N = 4 biological replicates for each condition. (PDF 147 kb) [file 13064_2018_121_MOESM9_ESM.pdf]

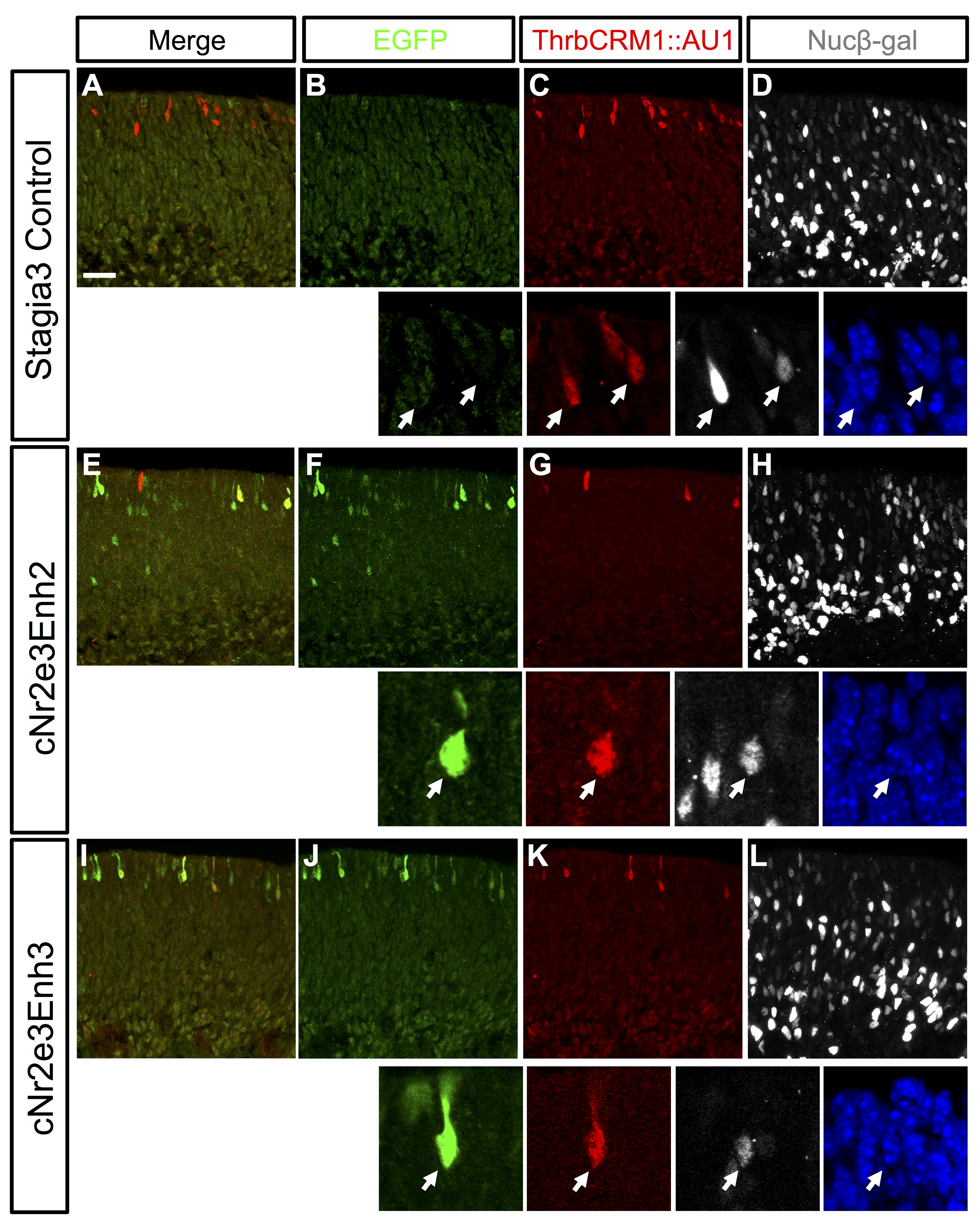

Supplement: Supplementary file 10 — Distribution of cells with Nr2e3Enh2 and Nr2e3Enh3 activity in the embryonic mouse retina. Embryonic day 14.5 retinas were co-electroporated with the Stagia3 plasmid shown to the left, ThrbCRM1::AU1, and CAG::Nucβ-gal, cultured for 2 days and processed for immunofluorescence confocal imaging for EGFP, AU1, β-gal, and DAPI. Maximum projections of z-stacks are shown in panels A-L with the channel depicted at the top of the column. Merge contains signals from EGFP, AU1, and β-gal channels. Single z-planes are shown below in the order of EGFP, AU1, β-gal, and DAPI from left to right. Arrows point to the position of ThrbCRM1::AU1+, Rxrg+ cells in each picture. In each image, the retina is oriented with the scleral portion at the top. Scale bar in A is 20 μm and applies to panels A-L. (PNG 9369 kb) [file 13064_2018_121_MOESM10_ESM.png]
